# Characterization and Monitoring of Isomalto/Malto-Polysaccharide Formation by Different 4,6-α-Glucanotransferases

**DOI:** 10.1021/acs.jafc.5c07954

**Published:** 2025-10-02

**Authors:** Nele Brand, Oliver Müller, Daniel Wefers

**Affiliations:** Institute of Chemistry, Food Chemistry, 9176 Martin Luther University Halle-Wittenberg, 06120 Halle (Saale), Germany

**Keywords:** maltodextrin, starch modification, structural analysis, NMR spectroscopy, enzymatic fingerprinting, reaction monitoring

## Abstract

GtfB type I enzymes, a subfamily of 4,6-α-glucanotransferases, are enzymes which convert starch into isomalto/malto-polysaccharides (IMMPs) by synthesizing α-1,6-linked chains. The structure of IMMPs highly depends on the enzyme and the substrate used. In this study, the IMMP formation of eight GtfB type I enzymes was investigated in detail by using different substrates and conditions. ^1^H NMR spectroscopy was used to analyze the structural composition (including the portion of released glucose) and revealed little impact of pH and temperature on the product composition for the investigated enzymes. However, enzymatic fingerprinting analysis revealed enzyme-dependent differences in the length distribution of the 1,6-linked sections of the IMMPs. A detailed reaction monitoring by HPAEC-PAD and NMR spectroscopy showed that larger oligosaccharides are initially used for IMMP synthesis and that branched malto-oligosaccharides are also converted. Overall, detailed information on the reaction time course and the structural composition of the products were obtained.

## Graphical abstract

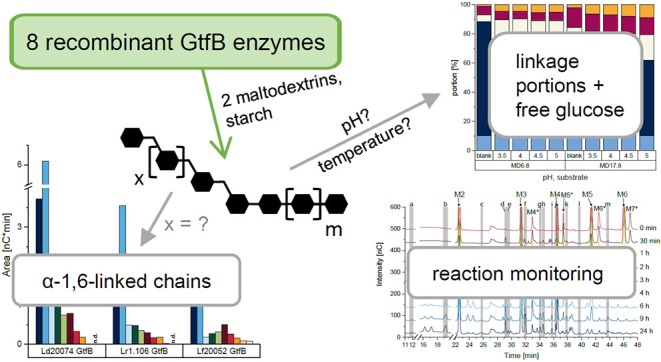

## 1 Introduction

Starch-modifying enzymes that produce dietary fiber, prebiotics, or other functional polysaccharides became more important in the last years, because they can be used to improve the nutritional and functional properties of starch. 4,6-α-glucanotransferases are starch-modifying enzymes from the GH70 family which display the evolutionary intermediate between the GH13 and the GH70 family.^1^ Those enzymes are able to remove the nonreducing end of an α-1,4-linked starch or maltodextrin fragment and transfer the glucosyl (or rarely maltooligosaccharyl) unit to the nonreducing end of an α-1,4-linked acceptor molecule. The repetition of this reaction results in the formation of (mostly) α-1,6-linked glucose chains. However, it is also possible that water acts as an acceptor resulting in the release of the glucosyl (or rarely maltooligosaccharyl) unit.^2^ Three different subfamilies of 4,6-α-glucanotransferases are known: GtfB, GtfC, and GtfD.^3,4^ Most of the already described enzymes belong to the GtfB subfamily which is further divided into GtfB type I and type II.^5^ GtfB type I enzymes transfer glucose units and form linear α-1,6-linked chains at the nonreducing end of α-1,4-linked acceptor molecules which results in reaction products named isomalto/malto-polysaccharides (IMMPs).^6–11^ In contrast, GtfB type II enzymes transfer malto-oligosaccharide units which results in the formation of branched, reuteran-like products.^5,11–16^ Interestingly, a 4,3-α-glucanotransferase from *Limosilactobacillus (Llb.) fermentum* NCC 2970 is known to form α-1,3-linkages instead of α-1,6-linkages.^17^

The products formed by GtfB type I enzymes highly depend on the substrate: Linear malto-oligosaccharides with a degree of polymerization (DP) > 6 were described to be good substrates for IMMP synthesis while the conversion of smaller malto-oligosaccharides (especially with a DP < 4) depends on the enzyme.^7,8,11,18,19^ The products formed from maltoheptaose were analyzed in most detail revealing products with a ratio of α-1,4-/α-1,6-linkages between 64:36 and 50:50 and a DP up to 35.^7,8,11,18^ In addition, the IMMPs occasionally contained intermediate α-1,4-linkages imbedded in the chains or at the nonreducing end of α-1,6-linked sections indicating a minor α-1,4-transglycosylation activity or an endolytical activity.^7,18^

The conversion of maltodextrins results in products with lower portions of 1,6-linkages (24–36%)^2,10,20^ which is most likely caused by the presence of branched oligo- and polymers. The presence of two loops (A1 and B) in GtfB type I enzymes that form a tunnel-like structure over the donor site of the binding groove results in a much less effective conversion of substrates that contain branches at position *O*6.^1,2,20^ The less effective modification can also be observed for most starches, whose conversion highly depends on the amylose/amylopectin content. In contrast, most GtfB type II enzymes have a more open active site due to shorter loops A1 and B. This eventually results in a much better conversion of branched substrates.^21^

Therefore, amylose and debranched starches are the best substrates for GtfB type I enzymes and can be converted to products with up to 92% α-1,6-linkages.^20^ IMMP synthesis can be further improved by adding isomalto- or malto-oligosaccharides.^2,22^ However, the amount of glucose released during IMMP synthesis was rarely analyzed. Glucose may be formed in significant amounts and vary from enzyme to enzyme, which was also suggested by the data provided by Bai et al.^2^

Therefore, our aim was to analyze all products formed by eight GtfB type I enzymes from different substrates under different reaction conditions. The enzymes selected for this study were derived from lactic acid bacteria which were able to fermentatively form IMMPs in culture media and sourdough.^23,24^ For the detailed analysis of the reaction products, their linkage portions including the portion of glucose that was released due to the hydrolytic activity of the enzymes were analyzed by ^1^H NMR spectroscopy. Based on the results, three enzymes were characterized in more detail. To obtain detailed insights into IMMP formation and IMMP structures, the fingerprinting method by van der Zaal et al.^25^ was applied. Furthermore, a detailed reaction monitoring by using NMR spectroscopy and high-performance anion exchange chromatography with pulsed amperometric detection (HPAEC-PAD) was carried out.

## 2 Materials and Methods

### 2.1 Materials

*Fructilactobacillus (Flb.) sanfranciscensis* DSM 20451, *Llb. panis* DSM 6035, *Llb. fermentum* DSM 20052, *Lactobacillus (Lb.) delbrueckii* subsp. *delbrueckii* DSM 20074, and *Lactiplantibacillus (Lpb.) argentoratensis* DSM 16365 were purchased from the German Collection of Microorganisms and Cell Cultures (DSMZ) GmbH, Braunschweig, Germany. *Llb. reuteri* TMW 1.106, *Flb. sanfranciscensis* TMW 1.1154, and *Flb. sanfranciscensis* TMW 1.2139 were kindly provided by Prof. Rudi Vogel and Prof. Fabio Minervini. If not stated otherwise, all chemicals used were of “p.a.” grade or better and were purchased from Carl Roth (Karlsruhe, Germany), Merck (Darmstadt, Germany), Thermo Fisher Scientific (Waltham, MA), VWR (Darmstadt, Germany), and Grüssing GmbH (Filsum, Germany). Two maltodextrins with dextrose equivalents (DE) of 6.8 (MD6.8) and 17.8 (MD17.8) were purchased from Merck (Darmstadt, Germany). Isopullulanase from *Aspergillus niger* (EC 3.2.1.57, 1000 U/mL), isoamylase HP from *Pseudomonas* sp. (EC 3.2.1.68, 500 U/mL), and β-amylase from barley (EC 3.2.1.2, 10000 U/mL) were purchased from Megazyme (Bray, Ireland).

### 2.2 Molecular Cloning and Heterologous Expression

First, genomic DNA was extracted from the cultures of the individual strains in modified Spicher medium^23^ by using the E.Z.N.A. Bacterial DNA kit (Omega Bio-Tek Inc., Norcross, GA) as described by Münkel et al.^26^ Gene sequence and ORF were derived from the literature (Lr1.106 GtfB, described by Kaditzky et al.^27^) or identified by analysis of the whole genome (accession numbers: Table S1) using BLAST (basic local alignment search tool) with the sequence of the GtfB of *Llb. reuteri* 121 (Lr121 GtfB, GenBank accession no. AAU08014.2)^28^ as query sequence. Additionally, BLAST searches were used to assess whether an *N*-terminally truncated variant of the individual glucanotransferases occurs in other bacteria. If this was the case, the individual glucanotransferases were truncated accordingly to enhance heterologous expression. The DNA fragments encoding for the 4,6-α-glucanotransferases were amplified from genomic DNA by using the Phusion High-Fidelity PCR kit (Thermo Fisher Scientific) and the primers listed in Table S2. For insertion of the gene into the pLIC-SGC1 vector via ligation independent cloning (LIC), the primers used for amplification included specific overhangs (Table S2). Amplified DNA was purified by using the MicroElute Cycle-Pure kit (Omega Bio-Tek Inc.) and cloned into the pLIC-SGC1 vector by using LIC.^29^ Molecular cloning, heterologous expression, and protein purification was carried out as described by Münkel et al.^26^ with minor modifications. Briefly, the gene-vector annealing products were transformed into 5α competent *Escherichia* (*E.*)­*coli* (High Efficiency) (NEB, Ipswich, MA) by heat shock at 42 °C, the cells were cultured at 37 °C, and the plasmids were isolated by the E.Z.N.A. Plasmid DNA Mini kit (Omega Bio-Tek Inc.). Sequences were confirmed by Sanger sequencing (Eurofins GATC Biotech, Konstanz, Germany). For heterologous expression, the plasmids were transformed into BL21 Star. First, a single colony was cultivated in 25 mL LB medium with 100 μg/mL ampicillin at 37 °C and 225 rpm for 16 h. This preculture was used to inoculate 800 mL of LB medium with 100 μg/mL ampicillin which was incubated under the same conditions until an OD600 between 0.5 and 0.9 was reached. Subsequently, gene expression was induced by adding IPTG (isopropyl-β-d-thiogalactopyranoside) to a final concentration of 0.1 mM, and incubation was continued at 20 °C and 225 rpm for 16 h. After that, cells were recovered by centrifugation and cell lysis was carried out in 50 mM phosphate buffer (with 300 mM sodium chloride, pH 7.5) by sonification (3 × 20 s pulse, 59.9 s pause, 50% amplitude) with an SFX250 sonifier (Branson Ultrasonics Corporation, Brookfield, CT). The lysate was centrifuged and the supernatant was added to a HisPur Ni-NTA resin (Thermo Fisher Scientific) for protein purification. Subsequently, the resin was incubated with the supernatant at 4 °C for 30 min, washed three times with wash buffer (10 mM imidazole, 50 mM sodium phosphate buffer, 300 mM sodium chloride, pH 7.5), and proteins were eluted with elution buffer (250 mM imidazole, 50 mM sodium phosphate buffer, 300 mM sodium chloride, pH 7.5). The obtained proteins were named according to their respective strain and used for further analyses (Table 1).

**Table 1:** Descriptors and Accession Numbers of the Recombinant 4,6-α-Glucanotransferases Which Were Analyzed in This Study

bacterial strain	4,6-α-glucanotransferase	accession number
*Lactobacillus delbrueckii* subsp. *delbrueckii* DSM 20074	Ld20074 GtfB	KRK22073.1
*Limosilactobacillus reuteri* TMW 1.106	Lr1.106 GtfB	ABP88725.1
*Limosilactobacillus fermentum* DSM 20052	Lf20052 GtfB	EEI21226.1
*Limosilactobacillus panis* DSM 6035	Lp6035 GtfB	KRM25865.1
*Fructilactobacillus sanfranciscensis* DSM 20451	Fs20451 GtfB	KRM78746.1
*Fructilactobacillus sanfranciscensis* TMW 1.2139	Fs1.2139 GtfB	WP_180993553.1
*Fructilactobacillus sanfranciscensis* TMW 1.1154	Fs1.1154 GtfB	NDR70316.1
*Lactiplantibacillus argentoratensis* DSM 16365	La16365 GtfB	KRL97820.1

### 2.3 Enzyme Activity and IMMP Synthesis

To use a reproducible enzymatic activity for IMMP synthesis, the hydrolytic activity of the 4,6-α-glucanotransferases was determined by monitoring the hydrolysis of 5 mM *para*-nitrophenyl-α-glucoside in sodium acetate buffer (25 mM with 1 mM CaCl_2_, pH 4.0 for Ld20074 GtfB and La16365 GtfB, pH 4.5 for Lr1.106 GtfB and Fs20451 GtfB, or pH 5.0 for Lf20052 GtfB, Lp6035 GtfB, Fs1.1154 GtfB, and Fs1.2139 GtfB) at 37 °C in a microplate reader (Infinite 200 Pro, Tecan, Männedorf, Switzerland) at 400 nm. The activity in U (μmol of released *para*-nitrophenol/min) was determined by using a calibration of 0.05 to 5 mM nitrophenol and a time interval of 60–120 min. However, it needs to be emphasized that the hydrolytic activity on this artificial substrate does not provide any information on the actual transferase activity and cannot be used to compare different enzymes among each other. It is solely used to ensure the same amount of active enzyme is added to the reaction mixture. If not stated otherwise, IMMP synthesis was carried out in sodium acetate buffer (25 mM with 20 mg/mL MD6.8 and 1 mM CaCl_2_) with 1 mU GtfB/mg maltodextrin at 37 °C and pH 4.0 for Ld20074 GtfB, pH 4.5 for Lr1.106 GtfB, or pH 5.0 for Lf20052 GtfB for 24 h. After the incubation, enzymes were inactivated at 95 °C for 15 min and removed by centrifugation at 21,714*g* for 15 min.

### 2.4 NMR Analysis of the Reaction Mixtures

The lyophilized reaction mixtures were dissolved in D_2_O (20 mg/mL) and acetone was added as a reference (referenced to 2.22 ppm according to Gottlieb et al.^30^). Samples were analyzed on a 400 MHz VNMRS, a 600 MHz VNMRS, or a 500 MHz DD2 spectrometer (Agilent, Santa Clara, CA) at 302 K. ^1^H NMR spectra were recorded with at least 32 scans. The 2D NMR experiments (HSQC, HMBC, and HSQC-TOCSY) were carried out on a 600 MHz VNMRS spectrometer. 32 scans were recorded for the HSQC and HMBC spectra. For the HSQC-TOCSY spectrum, 64 scans and a mixing time of 180 ms were used.

### 2.5 Fingerprint Analysis

Fingerprint analysis of the IMMPs present in the reaction mixtures was performed as described by van der Zaal et al.^25^ with minor modifications. The freeze-dried residues obtained from the incubation with the recombinant glucanotransferases were first dissolved in sodium acetate buffer (20 mM NaOAc with 5 mM CaCl_2_, pH 5.5; concentration: 2.5 mg/mL). Subsequently, 0.8 U isoamylase HP, 2 U β-amylase, and 0.8 U isopullulanase were added to 1 mL sample solution and the mixture was incubated at 40 °C for 4 h. To terminate the reaction, enzymes were inactivated at 95 °C for 10 min and removed by centrifugation (4 °C, 21,750*g*, 20 min). Hydrolyzed samples were diluted and analyzed by HPAEC-PAD on an ICS-6000 system (Thermo Fisher Scientific, Waltham, MA) equipped with a Carbo-Pac PA200 column (250 mm × 3 mm i.d., 5.5 μm particle size, Thermo Fisher Scientific). The column temperature was 30 °C and the detector temperature 25 °C. The following gradient was used with a flow rate of 0.4 mL/min: Column equilibration for 20 min with 10 mM NaOH, 0–10 min: isocratic with 10 mM NaOH, 10–20 min: linear gradient from 10 mM NaOH to 105 mM NaOH, 20–85 min: linear gradient from 105 mM NaOH to 105 mM NaOH + 200 mM NaOAc, 85–95 min: linear gradient from 105 mM NaOH + 250 mM NaOAc to 200 mM NaOH + 500 mM NaOAc, 95–110 min: isocratic with 200 mM NaOH + 500 mM NaOAc, 110–125 min: isocratic with 200 mM NaOH. The untreated maltodextrins were analyzed under the same conditions and used as control. To identify the linear α-1,6-linked isomalto-oligosaccharides in the chromatogram, the linear dextran synthesized by the truncated dextransucrase of *Ligilactobacillus animalis* TMW 1.971 (LaniDSΔN)^31,32^ was partially hydrolyzed with 0.05 M trifluoroacetic acid at 100 °C for 2 h and used as a reference mixture.

### 2.6 Reaction Monitoring

To analyze the course of the glucanotransferase reaction, 400 mg of MD17.8 were dissolved in 20 mL of sodium acetate buffer (25 mM with 1 mM CaCl_2_, pH 4.0, 4.5, or 5.0). The solution was incubated with 400 mU of Lr1.106 GtfB, Ld20074 GtfB, or Lf20052 GtfB at 40 °C for 24 h. Samples of 1 mL were taken at the beginning of the incubation, after 15 min, 30 min, 45 min, 60 min, 90 min, 120 min, 150 min, 3, 4, 5, 6, 7, 8, 9, and 24 h. Enzymes were inactivated immediately after taking the sample by heating them to 95 °C for 15 min and removed by centrifugation at 21,714*g* for 15 min. 750 μL of the supernatant were lyophilized and subsequently analyzed by ^1^H NMR spectroscopy as described above. The rest of the supernatant was diluted and analyzed by HPAEC-PAD as described in section 2.5. To identify some of the peaks in the chromatograms, linear malto-oligosaccharides with a DP of 2 to 7 were used as acceptors for the dextransucrase LaniDSΔN.^32^ For this, a 30 mM sucrose solution in sodium acetate buffer (50 mM with 1 mM CaCl_2_, pH 6.0) was incubated with the respective malto-oligosaccharide (final concentration: 30 mM) and 60 mU of LaniDSΔN at 40 °C for 6 h. Subsequently, the enzyme was inactivated at 95 °C for 15 min, the reaction mixture was diluted, and used as a reference for HPAEC-PAD analysis. To identify the DP of the standards, the HPAEC-PAD system was coupled to an LTQ-XL linear ion trap mass spectrometer (Thermo Fisher Scientific). Simultaneous PAD and MS analysis was allowed by a post column split. Prior to MS analysis, the eluent was desalted by an AERS 500e suppressor (4 mm, Thermo Fisher Scientific). To enable ionization by electrospray ionization (ESI), 500 μM LiCl was added to the eluate from the suppressor at a flow rate of 0.05 mL/min (AXP-MS pump, Thermo Fisher Scientific). Source temperature was 300 °C and lithium adducts were detected in positive mode.

## 3 Results and Discussion

### 3.1 Sequence Alignment and Heterologous Expression

The eight GtfB type I enzymes investigated in this study were selected based on our studies on the fermentative synthesis of IMMPs.^23,24^ The lactic acid bacteria encoding the corresponding genes were able to fermentatively synthesize significant portions of α-1,6-linked chains from maltodextrin. Therefore, the respective enzymes were selected to analyze enzymatic IMMP formation in detail. The sequences of these 4,6-α-glucanotransferases were derived from the literature (Lr1.106 GtfB^27^) and were identified by a BLAST Search of the genomic DNA with the GtfB from *Llb. reuteri* 121 (accession numbers are shown in Table 1). Sequence identity analysis revealed some high similarities (>95%) between enzymes of our study and those from literature but also GtfB enzymes that are not similar to all of the already described 4,6-α-glucanotransferases (e.g., Lp6035 GtfB) (Table S3). However, the homologues investigated in our study have not been reported yet. Comparison of the domain architecture of all eight GtfB enzymes showed a similar architecture for all GtfB enzymes. The most notable difference was the length of the *N*-terminal domain (Figure S1). However, it was already described that this domain does not affect the structure of the core of the protein or IMMP formation.^1,2^ Sequence alignment showed similarities of 49.93 to 97.61% between the GtfB enzymes (Table 2). Furthermore, the sequences of the conserved motifs I–IV and loops A1, A2, and B were analyzed (Figure S2). Most enzymes showed similarities between 50 and 70% and also some differences in the conserved motifs and the sequences of the loops. Notably, Fs20451 GtfB has a comparatively low similarity to the other GtfB enzymes, significantly shorter loops B and A1, as well as some mutations in the conserved motifs II, III, and IV that were not identified in the other sequences. In contrast, Lr1.106 GtfB has the highest similarity to Lr121 GtfB (92.48%) with identical motifs I–IV and only some differences in the sequence of loops B, A1 and A2. Furthermore, Ld20074 GtfB and La16365 GtfB have the highest similarity to each other (97.61%). However, both enzymes show differences in motif II (V in Ld20074 GtfB and I in La16365 GtfB at position 1014, Figure S2) as well as some variations of the amino acids in the sequences of the loops (also see Figure S2).

**Table 2:** Sequence Identity of the Amino Acid Sequences of GtfB from *Llb. reuteri* 121 (Lr121) and the Eight GtfB Enzymes Investigated in This Study in %^a^ ^37^

	Lr121	Lr1.106	Lf20052	Ld20074	Lp6035	La16365	Fs20451	Fs1.1154	Fs1.2139
Lr121	100	92.48	65.62	62.92	64.29	83.01	57.09	71.93	50.51
Lr1.106	92.48	100	65.52	62.76	65.62	82.78	57.09	71.25	53.65
Lf20052	65.62	65.52	100	67.27	63.14	72.74	66.80	70.70	69.21
Ld20074	62.92	62.76	67.27	100	61.92	97.61	58.87	71.80	57.02
Lp6035	64.29	65.62	63.14	61.92	100	78.36	56.73	69.53	49.93
La16365	83.01	82.78	72.74	97.61	78.36	100	70.62	71.69	71.46
Fs20451	57.09	57.09	66.80	58.87	56.73	70.62	100	89.24	92.41
Fs1.1154	71.93	71.25	70.70	71.80	69.53	71.69	89.24	100	95.28
Fs1.2139	50.51	53.65	69.21	57.02	49.93	71.46	92.41	95.28	100

a The strains corresponding to the enzymes as well as the accession numbers of the sequences used for comparison are shown in Table 1. Sequence identity was determined by using the EMBL-EBI Job Dispatcher sequence analysis tools framework from Clustal Omega.^37^

To gain insights into the activity and reaction products of the GtfB enzymes, the corresponding genes were cloned into the pLIC-SGC1 vector and heterologously expressed in *E. coli*. The individual enzymes were then used to form IMMPs from maltodextrins and the reaction mixtures were characterized by using ^1^H NMR spectroscopy.

### 3.2 Assignment of NMR Signals

To gain insights into the transglycosylation activity of the GtfB enzymes, the portions of α-1,4-, α-1,4,6- and α-1,6-linked glucose units in the reaction products were determined by integrating the signals of the respective anomeric protons in the ^1^H NMR spectra (Figure 1). However, it has to be considered that the first glucose unit of side chains present in maltodextrins (units H and J in Figure 1) gives the same signal as linear α-1,6-linked glucopyranoses and is therefore included in the portion of α-1,6-linked glucose units. Furthermore, the signals for glucose units at the reducing end of oligo- or polysaccharides as well as the signals for free glucose were integrated (Figure 1). The latter can also be used to obtain information on the hydrolytic activity. To distinguish between these two types of reducing glucose units, we compared the ^1^H NMR spectra of IMMPs with those of glucose and maltose. While the signals of the reducing α-configurated glucose units completely overlapped, the doublets of the reducing β-glucose units showed only partial overlap: the doublet signal at 4.65 ppm can be assigned to the anomeric proton of a glucose unit at the reducing end of an oligo- or polysaccharide, whereas the doublet signal at 4.63 ppm belongs to the anomeric proton of free β-glucose (Figure 1). By integration of the nonoverlapping doublet parts, the ratio between the two types of reducing glucose units can be determined. This ratio was combined with the sum of all integrals from reducing glucose units to calculate the portions of free glucose and glucose units at the reducing end of oligo- or polysaccharides.

**Figure 1:**
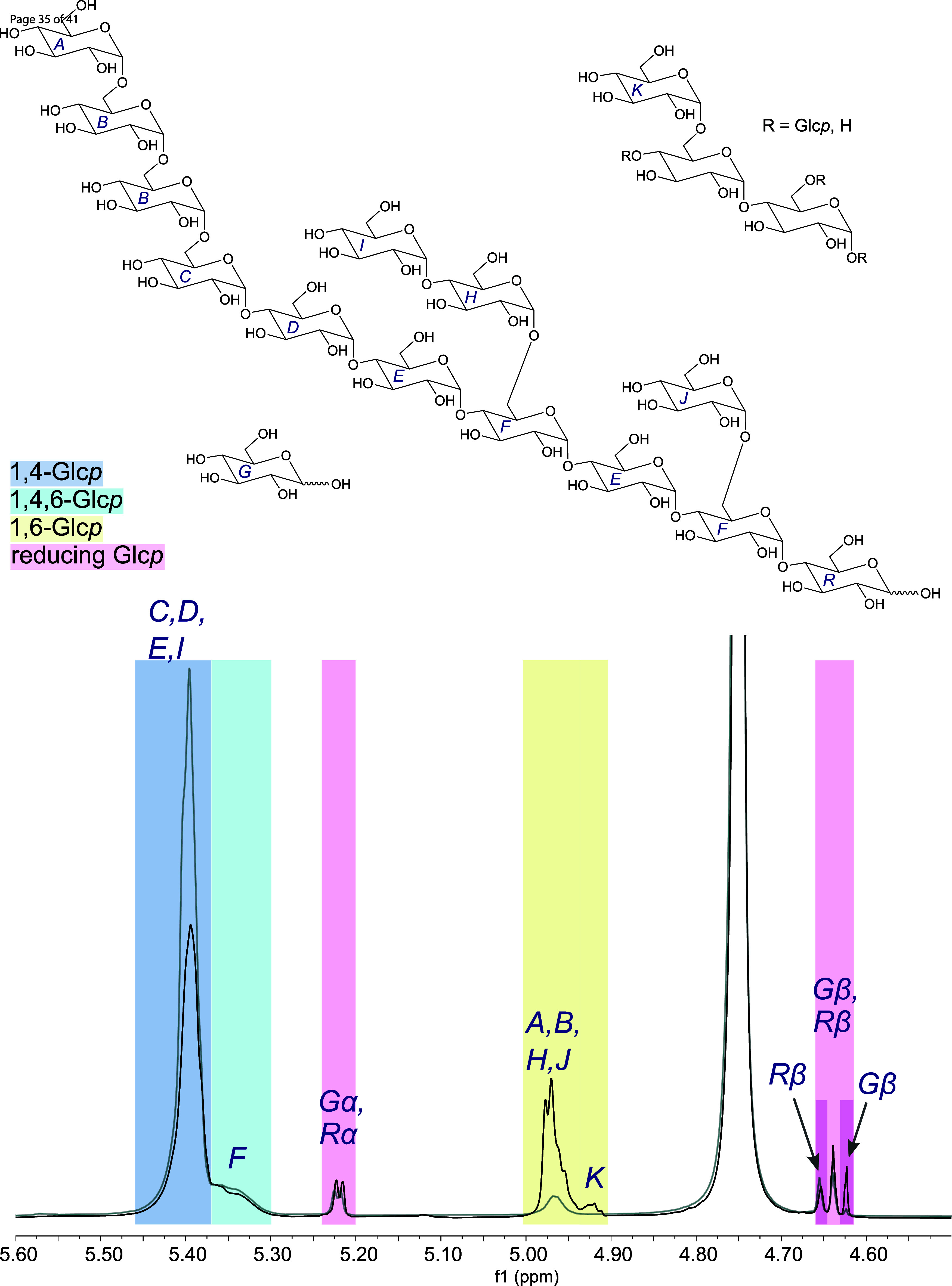
Assignment of the anomeric signals in the ^1^H NMR spectra of the reaction products synthesized by Lr1.106 GtfB from maltodextrin with a dextrose equivalent of 6.8 (MD6.8) and pH 5 at 37 °C (black) compared to the respective maltodextrin (gray).

In addition, the signal with a chemical shift of 4.92 ppm in the ^1^H NMR spectra of several GtfB reaction products (unit K in Figure 1) has not been described in the literature and was not found in the ^1^H NMR spectra of glucose, maltotriose, isomaltotriose, panose, maltodextrin, or mixed-linkage reuteran-like glucans.^33^ The signal increases during the course of IMMP formation which indicates that it represents a structural feature formed by GtfB enzymes. Notably, the signal was not present in the ^1^H NMR spectra of the fermentatively synthesized IMMPs,^23^ demonstrating that the compounds containing the unknown structural element were removed by the applied ethanol precipitation. This indicates that the structural unit belongs to an oligosaccharide or to a polysaccharide with a very low molecular weight. Because the signal has a comparatively high intensity in the ^1^H NMR spectrum of the reaction mixture obtained from the incubation of maltoheptaose with Lr1.106 GtfB, HSQC, HMBC and HSQC-TOCSY experiments were carried out to obtain more information about the underlying structural element (Figures S3–S5). The chemical shifts of the anomeric signal obtained from the HSQC spectrum (Figure S3) (^1^H: 4.92 ppm, ^13^C: 98.8 ppm) indicate that the signal represents a glucose unit that is linked to position *O*6 of the neighboring glucose unit, because the anomeric protons/carbons involved in this linkage type usually have chemical shifts between 4.95–5.00 ppm (^1^H) and 98–99 ppm (^13^C).^18,26,33–35^ This is confirmed by the HMBC spectrum, which showed a correlation of the anomeric proton to a ^13^C signal at 69.3 ppm (Figure S4). Two ^13^C signals at 69.3 ppm (^1^H chemical shifts: 3.76 and 3.58 ppm; Figure S3) were detected in the HSQC spectrum, therefore, they can be assigned to the C6H6 signal of a glucose unit which yields two signals in the proton dimension due to the two diastereomeric protons attached to C6. By using the HSQC-TOCSY spectrum (Figure S5), the ^13^C signal at 69.3 ppm can be assigned to a C6 signal of a glucopyranose unit that is itself linked to position *O*-4 of the following unit. This can be derived from the correlation of the H6 protons with the ^13^C signal at 100.3 ppm which are characteristic for 1,4-linked glucose units (typically at 100.3–100.6 ppm). In addition, the HSQC-TOCSY spectrum showed a correlation of the anomeric proton of unit K at 4.92 ppm with the carbons at 70.0 and 61.0 ppm. These chemical shifts are characteristic for an unsubstituted C4 and C6, respectively. Therefore, the results from the NMR spectroscopic characterization suggest that the structural unit has to be located at the nonreducing end and that it is linked to position *O*6 of the following glucose unit, which is in turn linked to the position *O*-4 of a third glucose unit [**Glc*p*-(α1** → 6)-Glc*p*-(α1 → 4)-Glc*p*-(α1 →···)]. However, the signal was not observed in panose or maltodextrins. Therefore, the second glucose unit may be branched or it may be bound to position *O*-4 of a branched unit. However, although we were not able to unambiguously identify all neighboring units of unit K, the signal is clearly derived from the formation of an α-1,6-linkage by GtfB enzymes. Therefore, the integral of the corresponding signal was included into the calculation of the portion of α-1,6-linked glucopyranose units.

### 3.3 Characterization of the Reaction Mixtures Obtained from the GtfB Enzymes

The evaluated NMR signals were then used to analyze the composition of the reaction mixtures after incubation with the GtfB enzymes. Two maltodextrins with different dextrose equivalents (MD6.8 and MD17.8) were used to enzymatically synthesize IMMPs at different pH (3.5, 4.0, 4.5, and 5.0) and different temperatures (25, 30, and 37 °C). These ranges were chosen because most GtfB type I enzymes have a high activity under these conditions.^6–8^ Figure 2 shows the structural composition of the products synthesized by Lr1.106 GtfB from MD6.8 and MD17.8 at different pH values. In all reaction products, the portions of α-1,6-linked glucose units and monomeric glucose are significantly higher than in the respective untreated maltodextrins. The clearly higher abundance of the 1,6-linkages compared to monomeric glucose suggests that the enzyme preferred transglycosylation over hydrolysis in all reaction mixtures. As expected, the portion of α-1,4-glucosidic linkages decreased, while the portion of 1,4,6-linked glucose units and glucose units at reducing ends remained constant. The products obtained from the incubation of MD6.8 contained higher portions of α-1,6-linkages (21.4–26.1%) compared to the products from MD17.8 (14.5–20.3%). The portions of the different structural elements align well with the results described for other GtfB enzymes from *Llb. reuteri*: 24–36% α-1,6-glucosidic linkages were obtained from different maltodextrins converted by Lr121 GtfB and 32% α-1,6-linkages were formed by Lr20016 GtfB (GtfW).^2,20^ In addition, FsTMW11304 GtfB formed 35% α-1,6-linkages from maltodextrin with a dextrose equivalent of 4.0–7.5.^10^ Regardless of the pH applied for the incubation, the structural composition of the products synthesized by Lr1.106 GtfB remained approximately the same which suggests only a minor influence of different pH values. In contrast, other studies showed a much higher impact of the pH on the activity of GtfB type I enzymes.^2,9^

**Figure 2:**
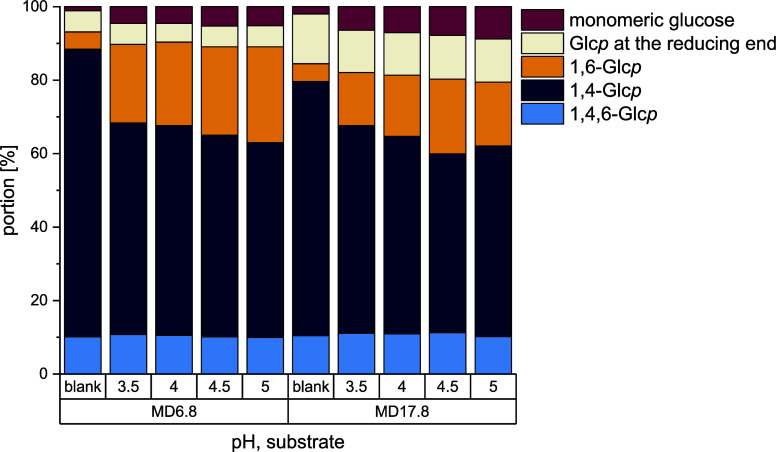
Portions of differently linked glucopyranoses (Glc*p*) and monomeric glucose in the reaction mixtures obtained by the incubation of maltodextrins with a dextrose equivalent of 6.8 (MD6.8) and 17.8 (MD17.8) with the GtfB from *Llb. reuteri* TMW 1.106 (Lr1.106 GtfB). All samples were incubated at 37 °C and different pH values for 24 h. The structural composition of the mixtures was analyzed by ^1^H NMR spectroscopy.

Similar results as for Lr1.106 GtfB were obtained for Ld20074 GtfB and Lf20052 GtfB (Figures S6 and S7). However, Fs1.1154 GtfB, Fs1.2139 GtfB, Fs20451 GtfB, La16365 GtfB, and Lp6035 GtfB only formed minor portions of α-1,6-glucosidic linkages from both maltodextrins (Figures S8–S12), although a hydrolytic activity was present (indicated by the portions of monomeric glucose). Interestingly, La16365 GtfB also showed weak transglycosylation activity despite the high sequence similarity to Ld20074 GtfB (Table 2). Furthermore, the portion of α-1,4,6-linked glucose units increased in the reaction products of Fs20451 GtfB (Figure S10) although the fermentatively formed IMMPs did not contain higher portions of α-1,4,6-linked glucose units.^23^ As Fs20451 GtfB has smaller loops B and A1 (Figure S2) which is also described for most GtfB type II enzymes that are able to synthesize branches at position *O*6,^1,5^ some branched compounds with a low molecular weight might have been formed. However, Lr1.106 GtfB, Lf20052 GtfB, and Ld20074 GtfB clearly showed the highest transglycosylation activity and formed significant portions of α-1,6-glucosidic linkages. Therefore, the IMMPs as well as the IMMP formation of these enzymes were characterized in more detail.

First, the impact of incubation temperature on the structural composition of the reaction products was analyzed. For that, the GtfB enzymes were used to convert MD6.8 at 25 °C, 30 and 37 °C and the reaction products were analyzed by ^1^H NMR spectroscopy. The results are shown in Figure 3. Lr1.106 GtfB, Ld20074 GtfB and Lf20052 GtfB were all able to synthesize significant portions of α-1,6-linkages at all three temperatures (increase from 6.4% in MD6.8 to 20.5–25.5% in the reaction mixtures). However, the reaction temperature mostly had no clear impact on the structural composition of the reaction mixtures. Slight changes in the composition of the reaction mixtures obtained with Ld20074 GtfB and Lf20052 GtfB (higher portions of monomeric glucose, lower portions of 1,6-linkages) indicated a higher hydrolytic activity at increased temperatures, however, the changes were only minor. In the literature, both a high and low dependency of the enzyme activity on the temperature was described.^2,9^ Therefore, the impact of the incubation temperature on IMMP synthesis most likely depends on the enzyme.

**Figure 3:**
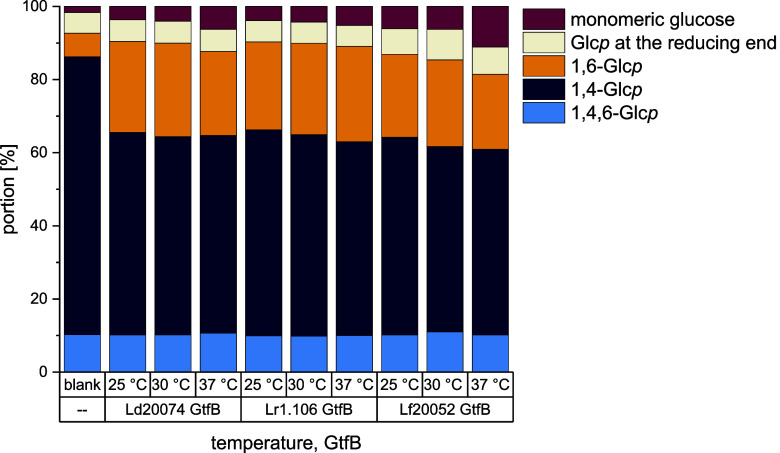
Portions of differently linked glucopyranoses (Glc*p*) and monomeric glucose in maltodextrin with a dextrose equivalent of 6.8 (blank) and the respective reaction mixtures obtained by the incubation of the maltodextrins with the 4,6-α-glucanotransferases from *Lb. delbrueckii* subsp. *delbrueckii* DSM 20074 (Ld20074 GtfB), *Llb. reuteri* TMW 1.106 (Lr1.106 GtfB), and *Llb. fermentum* DSM 20052 (Lf20052 GtfB) at pH 4.5 (Ld20074 GtfB) or pH 5.0 (Lr1.106 GtfB and Lf20052GtfB) and different temperatures for 24 h. The structural composition of the mixtures was analyzed by ^1^H NMR spectroscopy.

It has been described that several GtfB type I enzymes form significant portions of α-1,6-glucosidic linkages from starch, amylose, or debranched starch.^1,2,20,21^ Therefore, wheat starch was incubated with Ld20074 GtfB, Lr1.106 GtfB, and Lf20052 GtfB, with and without the addition of isoamylase as debranching enzyme. The reaction products were analyzed by ^1^H NMR spectroscopy and the structural composition of the reaction mixtures is shown in Figure 4. All three GtfB enzymes synthesized α-1,6-linkages from wheat starch (increase from 5.9% in starch to 9.5–19.4% in the reaction products), but also released significant portions of glucose during the reaction (7.1–8.7%). When the initially present portion of 1,6-linkages is taken into account, the hydrolytic activity seems to exceed or match the transglycosylation activity. As expected, starch debranching with isoamylase resulted in significantly higher portions of α-1,6-linkages in the reaction products (38.7–45.8%), but the portion of free glucose and glucose units at the reducing end increased as well. However, the ratios of α-1,6-linkages/α-1,4-linkages in the reaction mixtures derived from the incubation of debranched starch are between 60:40 and 70:30 which is in agreement to those described for IMMPs in the literature (90:10 and 74:26 by Lr121 GtfB and GtfW,^2^ 85:15 by GtfY-ΔNΔC,^11^ 72:28 by Lf3057 GtfB,^9^ 60:40 by FsTMW11304 GtfB,^10^ all synthesized from amylose). Only a few studies also analyzed the portions of free glucose resulting from the GtfB reaction. Very different portions of released glucose were described for GtfW (23%) and Lr121 GtfB (8%) indicating that the hydrolytic activity highly depends on the enzyme.^2^ This is in good agreement with our data and the ^1^H NMR spectra provided in other studies that showed very different intensities for the respective signals.^9–11,36^ However, our results provide detailed insights into the structural composition of the reaction mixtures of Ld20074 GtfB, Lr1.106 GtfB, and Lf20052 GtfB. Furthermore, they demonstrate that these GtfB enzymes can be applied to increase the contents of nondigestible α-1,6-linkages, glucose, and smaller saccharides in wheat starch (hydrolysates).

**Figure 4:**
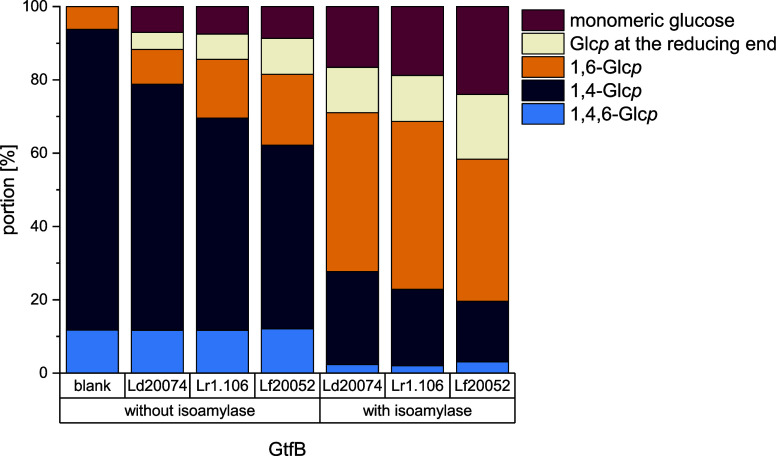
Portions of differently linked glucopyranoses (Glc*p*) and monomeric glucose in reaction mixtures obtained by the incubation of wheat starch with the GtfBs from *Lb. delbrueckii* subsp. *delbrueckii* DSM 20074 (Ld20074), *Llb. reuteri* TMW 1.106 (Lr1.106), and *Llb. fermentum* DSM 20052 (Lf20052), at 37 °C and pH 4.5 (Ld20074 GtfB) or pH 5.0 (Lr1.106 GtfB and Lf20052 GtfB) for 24 h, with or without the addition of isoamylase. The structural composition of the mixtures was analyzed by ^1^H NMR spectroscopy.

### 3.4 Fingerprint Analysis of the Products from Lr1.106 GtfB, Lf20052 GtfB and Ld20074 GtfB

To obtain more detailed information about the structure of the enzymatically synthesized IMMPs, the fingerprinting method developed by van der Zaal et al.^25^ was used. Because our particular interest was on the length distribution of the α-1,6-linked sections, the α-1,4-linked sections were completely hydrolyzed by using isopullulanase, isoamylase, and β-amylase, and the remaining α-1,6-linked chains were analyzed by HPAEC-PAD. For identification and size determination of the linear, α-1,6-linked sections, the linear dextran formed by the dextransucrase LaniDSΔN was partially hydrolyzed with trifluoroacetic acid and used as a size standard. HPAEC-PAD chromatograms of the fingerprints of the reaction mixtures obtained with MD6.8 and the three enzymes are shown in Figure S13. All three chromatograms showed 1,6-linked oligo- and polysaccharides with varying distributions as well as some additional peaks, which is in good agreement with the results obtained for the respective fermentatively synthesized IMMPs.^23^ To visualize the length distribution, the peak areas of selected linear, α-1,6-linked chains were used (Figure 5). Because the peak area of the PAD signals depends not only on the concentration but also on the DP, this approach does not describe a quantitative composition. However, Figure 5 clearly shows that Ld20074 GtfB, Lr1.106 GtfB, and Lf20052 GtfB synthesize 1,6-linked chains with varying size distributions: A higher portion of shorter α-1,6-linked chains (DP 2–15) can be detected when Ld20074 GtfB is used, while the reaction products of Lf20052 GtfB have significantly longer α-1,6-linked chains (DP > 50). This is in good agreement with the results obtained for the respective fermentatively synthesized IMMPs, because *Llb. fermentum* DSM 20052 also formed longer α-1,6-linked chains than *Lb. delbrueckii* subsp. *delbrueckii* DSM 20074 and *Llb. reuteri* TMW 1.106.^23^ Therefore, our results show that the three enzymes synthesize different products from the same substrate.

**Figure 5:**
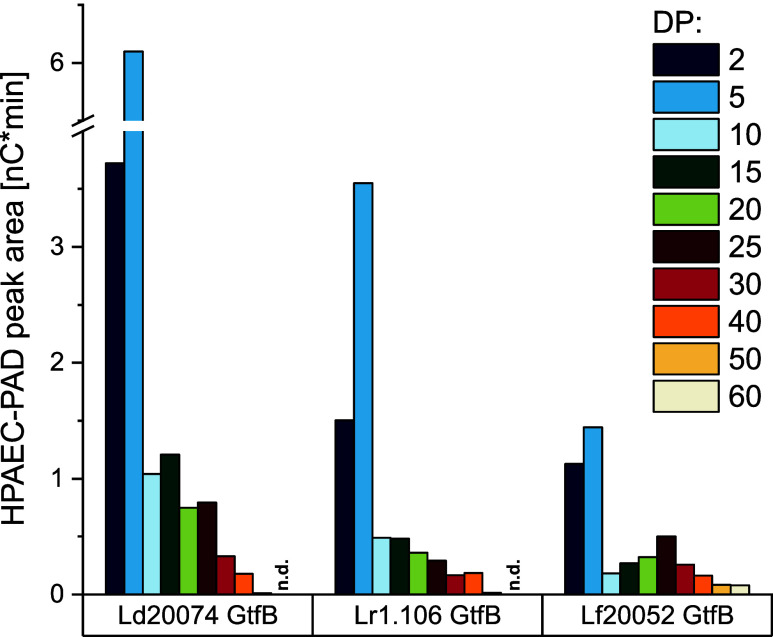
HPAEC-PAD peak areas of selected 1,6-linked oligo- and polysaccharides which were liberated by enzymatic hydrolysis of the reaction products formed by the GtfBs from *Lb. delbrueckii* subsp. *delbrueckii* DSM 20074 (Ld20074 GtfB), *Llb. reuteri* TMW 1.106 (Lr1.106 GtfB), and *Llb. fermentum* DSM 20052 (Lf20052 GtfB) from maltodextrin with a dextrose equivalent of 6.8. n.d. = not detected, DP = degree of polymerization..

### 3.5 Reaction Monitoring

To gain detailed insights into the formation of IMMPs and into the impact of reaction time on the product composition, the IMMP synthesis of the three enzymes was monitored over time. Thus, IMMPs were synthesized by incubating MD17.8 with Lr1.106 GtfB, Lf20052 GtfB, and Ld20074 GtfB, and samples were taken at various times of the reaction. After inactivation of the enzymes, the structural composition of the samples was analyzed by ^1^H NMR spectroscopy. Figure 6 shows the change of structural composition over time during IMMP synthesis by Lr1.106 GtfB. As expected, the portion of α-1,6-linked glucopyranoses as well as the portion of free glucose steadily increased over time, while the portion of α-1,4-linked glucopyranoses decreased. However, most α-1,6-linkages were formed during the first 9 h of the reaction as the increase between 9 and 24 h is comparatively small. Similar results were obtained for Lf20052 GtfB and Ld20074 GtfB (Figures S14 and S15).

**Figure 6:**
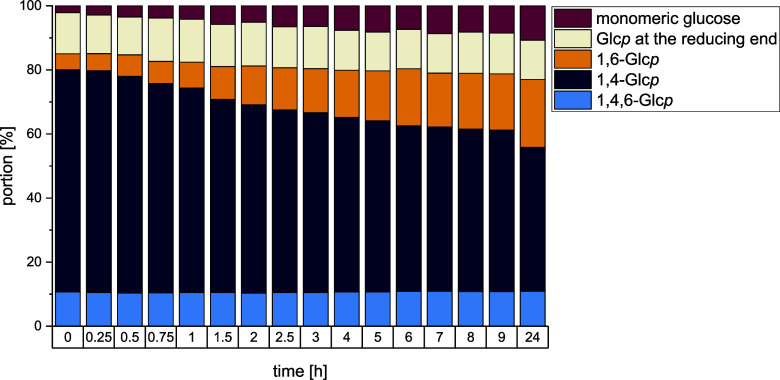
Portions of differently linked glucopyranoses (Glc*p*) and monomeric glucose at different times during the conversion of maltodextrin with a dextrose equivalent of 17.8 (MD17.8) by the GtfB from *Llb. reuteri* TMW 1.106. The reaction was carried out at pH 4.5 and 37 °C. The structural composition of the mixtures was analyzed by ^1^H NMR spectroscopy.

To obtain more information on the degradation and formation of low molecular weight reaction products during IMMP synthesis, HPAEC-PAD analysis was carried out. To identify some compounds, standards were synthesized from sucrose by using LaniDSΔN^32^ and different malto-oligosaccharides as acceptors. The homologous series of the respective malto-oligosaccharides elongated with α-1,6-linked glucopyranose units at the nonreducing end were identified by analyzing their DP and retention time with HPAEC-PAD/MS. In addition, linear isomalto-oligosaccharides synthesized by the hydrolysis of the linear dextran of LaniDSΔN^32^ were used as retention time standards. The HPAEC chromatograms of selected samples taken during IMMP synthesis by Lr1.106 GtfB are shown in Figure 7. All HPAEC chromatograms from the monitoring of the IMMP synthesis by Lr1.106 GtfB, Lf20052 GtfB, and Ld20074 GtfB are shown in Figures S16–S18. The chromatograms clearly show that malto-oligosaccharides with a higher DP are used as a substrate first, because the peaks of maltohexaose and maltopentaose are decreasing first. Interestingly, the peaks of the limit dextrins with a DP of 4–7 (M4*–M7*) decrease as well, demonstrating that those branched oligosaccharides can also be converted by Lr1.106 GtfB. The signal of the smallest limit dextrin shown in the chromatograms (M4*, *t*_R_ = 33 min) initially increases before it decreases which may be a result of the hydrolysis of larger limit dextrins and a subsequent conversion of M4*. Most likely, the different limit dextrins are used as donors and acceptors which could also explain the additional peaks observed in the enzymatic fingerprinting (e.g., peaks at 16–17, 24–35, 27–28, 38–39 and 41 min). Although some other studies reported mainly hydrolytic activities during the first hour of IMMP synthesis,^6,18^ our results unambiguously show the formation of transglycosylation products during the first 30 min of the reaction. For example, isomalto-oligosaccharides with a DP of 2 to 5 (peaks a, b, c, and e in Figure 7) were identified in the chromatograms indicating that glucose can be used as acceptor substrate by GtfB enzymes which is in agreement with other studies.^2,11,19^ However, isomaltose may also act as an acceptor substrate resulting in isomalto-oligosaccharides with a DP ≥ 3.^12^ In addition, several compounds of the homologous series of panose were identified (peaks d, f, g, and i in Figure 7) although the enzyme was not active on maltose itself (data not shown). Interestingly, these compounds were already formed at the beginning of the reaction despite our observation that larger malto-oligosaccharides are converted first. The same applies to the identified oligosaccharides containing maltotriose at the nonreducing end (peaks h, j, k, and l in Figure 7). Therefore, the enzymes might prefer larger oligosaccharides as donors, but maltose or maltotriose as acceptors. A signal of a glucose unit linked to position *O*6 of the nonreducing end of maltopentaose slowly increases and subsequently decreases after approximately 4 h which most likely results from its use as an acceptor. Further IMMPs with α-1,6-linked chains linked to malto-oligosaccharides with a DP ≥ 4 as described for example by Dobruchowska et al.^18^ are probably also present. However, those compounds were very difficult to identify under the conditions used due to decreasing detector responses and decreasing resolution/coelution of several compounds. The same applies for IMMPs with longer α-1,6-linked chains. Furthermore, some peaks with varying portions during the course of the reaction could not be assigned. Those peaks may be derived from compounds formed from limit dextrins, as those described by Leemhuis et al.^20^ In addition, it is possible that the GtfB enzymes occasionally transfer maltose or maltotriose units due to an endolytic activity.^1,19^ Furthermore, the enzymes may have an α-1,4-transglycosylation activity leading to IMMP structures with α-1,4-linked glucose units within the α-1,6-linked chains.^7,18^ The chromatograms obtained from the reactions of Ld20074 GtfB and Lf20052 showed the same trends, although some variations in the intensities of the individual compounds and the time course of the reactions could be observed. This once again demonstrates the similarities and differences between the enzymes investigated in this study.

**Figure 7:**
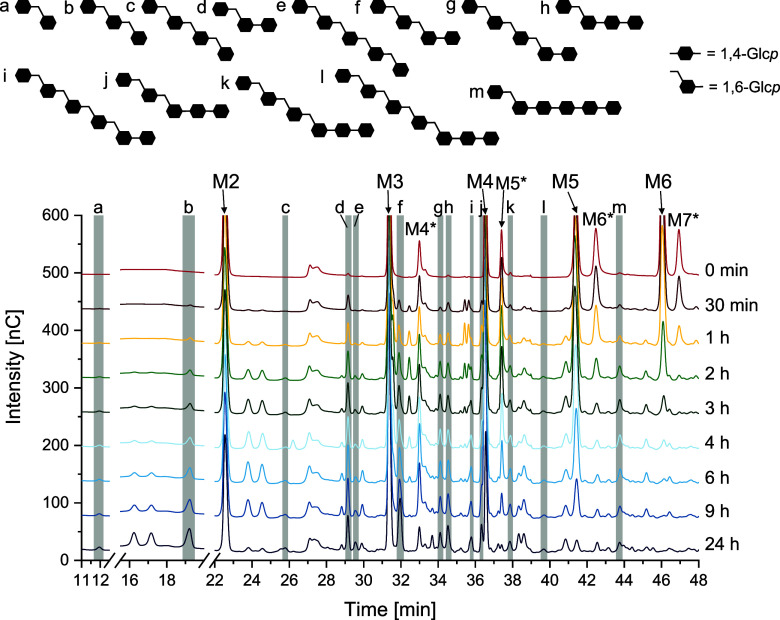
HPAEC-PAD chromatograms of selected samples taken during IMMP synthesis by the *Llb. reuteri* TMW 1.106 GtfB from maltodextrin with a dextrose equivalent of 17.8. M2 = maltose, M3 = maltotriose, M4 = maltotetraose, M5 = maltopentaose, M6 = maltohexaose, M4*–M7* = limit dextrins with the respective degree of polymerization..

Concluding, we obtained detailed information on the IMMP formation by eight GtfB enzymes and on the product structures of three selected enzymes. The structural composition of the reaction mixtures and the fine structures of the IMMPs were mainly influenced by the GtfB enzyme and the substrate used for the synthesis, while reaction conditions such as pH and temperature had no significant impact. Although relatively similar structural compositions were determined by NMR spectroscopy, enzymatic fingerprinting analysis revealed significant differences between the IMMPs formed by Lr1.106 GtfB, Ld20074 GtfB, and Lf20052 GtfB. Furthermore, we were able to gain some detailed insights into the time course of the IMMP formation. The conversion of linear and branched malto-oligosaccharides as well as the formation of several transglycosylation products demonstrated the complexity of the reaction products formed from maltodextrins. Especially the use of different malto-oligosaccharides as donors and acceptors, and the conversion of limit dextrins could be part of future investigations.

## Supplementary Material



## Notes

Accession numbers of the genome sequencing projects of the eight lactic acid bacteria, sequences of the PCR primers used, domain structures of the GtfB enzymes, amino acid sequences of conserved motifs and loops of the GtfBs, sequence identity of the GtfB enzymes described in literature and the eight GtfB enzymes from this study, 2D NMR spectra of IMMPs synthesized from maltoheptaose, structural compositions of the IMMPs synthesized by Lf20052 GtfB, Ld20074 GtfB, Lp6035 GtfB, La16365 GtfB, Fs20451 GtfB, Fs1.1154 GtfB, and Fs1.2139 GtfB at different pH values, HPAEC-PAD chromatograms from the enzymatic fingerprinting of the IMMPs formed by Lf20052 GtfB, Ld20074 GtfB, and Lr1.106 GtfB, monitoring of the structural compositions of the reaction products during the synthesis of IMMPs by Ld20074 GtfB and Lf20052 GtfB, HPAEC-PAD chromatograms from the reaction monitoring of IMMP formation by Lf20052 GtfB, Ld20074 GtfB, and Lr1.106 GtfB (PDF)

## Abbreviations

NTA: nitrilotriacetic acid
MD6.8: maltodextrin with a dextrose equivalent of 6.8
MD17.8: maltodextrin with a dextrose equivalent of 17.8
HPAEC-PAD: high-performance anion exchange chromatography with pulsed amperometric detection
*t*_R_: retention time
DP: degree of polymerization
ORF: open reading frames
LaniDSΔN: truncated dextransucrase from *Ligilactobacillus animalis* TMW 1.971
