# Supplementary material for: Characterization and Monitoring of Isomalto/Malto-Polysaccharide Formation by Different 4,6-α-Glucanotransferases
Source: J Agric Food Chem. 2025 Oct 2;73(41):26276–86. doi: 10.1021/acs.jafc.5c07954 (PMC12532295; doi:10.1021/acs.jafc.5c07954)
Supplement: Supplementary file 1 [file jf5c07954_si_001.pdf]

# Supporting Information

## Characterization and monitoring of isomalto/malto-polysaccharide formation by different 4,6-α-glucanotransferases

Nele Brand[a], Oliver Müller[a], Daniel Wefers[a*]

[a] Institute of Chemistry, Food Chemistry, Martin Luther University Halle-Wittenberg, 06120 Halle (Saale), Germany

**Tab. S1**: Accession numbers of the genome sequencing projects of the eight lactic acid bacteria whose 4,6-α-glucanotransferases were analyzed in this study.

| Bacterial strain | Accession number |
|---|---|
| *Limosilactobacillus reuteri* TMW 1.106 | - |
| *Limosilactobacillus fermentum* DSM 20052 | NZ_ACGI00000000 |
| *Lactobacillus delbrueckii* subsp. *delbrueckii* DSM 20074 | BALP00000000 |
| *Lactiplantibacillus argentoratensis* DSM 16365 | AZFR00000000 |
| *Limosilactobacillus panis* DSM 6035 | NZ_AZGM00000000 |
| *Fructilactobacillus sanfranciscensis* DSM 20451 | QRFO00000000 |
| *Fructilactobacillus sanfranciscensis* TMW 1.1154 | NZ_SCEU00000000 |
| *Fructilactobacillus sanfranciscensis* TMW 1.2139 | MIXZ00000000 |

**Tab. S2**: PCR primers used in this study (specific overhangs for ligation independent cloning are italicized).

| 4,6-α-glucano-transferase | Primer | Primer sequence (5' → 3') |
|---|---|---|
| LDEL04630c | Forward | *TACTTCCAATCCATG* AACGGTGACGCAATGGTCACCATC |
| LDEL04630c | Reverse | *TATCCACCTTTACTG* AAGTCGGGAAATTGAGATAACCGCTGCATG |
| LFER1834c | Forward | *TACTTCCAATCCATG* CAGACACATTTACGTGTAGATGATAATTATGTTCAATCGC |
| LFER1834c | Reverse | *TATCCACCTTTACTG* ATCGTCTTCAATATTAGCATAATAATCAGCGGAAGACATC |
| LREUgtfBc | Forward | *TACTTCCAATCCATG* AATACCTTAGTAAAAACATTAGGACCAGGTACTTG |
| LREUgtfBc | Reverse | *TATCCACCTTTACTG* ATCGTTGAAAATTGGTGCAATTTCACTTG |
| LPAN10260c | Forward | *TACTTCCAATCCATG* AATGGAGCAATGGTCACCATCCG |
| LPAN10260c | Reverse | *TATCCACCTTTACTG* ATTTTTTAATTCCTGTTGAACATAGTAATCAATCGATCCCATCTG |
| LARG0003 | Forward | *TACTTCCAATCCATG* TATGGCACAGATCATGTTCAGTATCGTAATCG |
| LARG0003 | Reverse | *TATCCACCTTTACTG* ATTTTTTAATTCCTGTTGAACATAGTAATCAATCGATCCCATC |
| LSAN00565c | Forward | *TACTTCCAATCCATG* TCTGATGGTCAATTATTGGGCAAAACTTATGGC |
| LSAN00565c | Reverse | *TATCCACCTTTACTG* AAGGTCAGCTTTATTCGCATAATAATCGACCG |
| LSAN03845c | Forward | *TACTTCCAATCCATG* AATAATAAATTCTACTACTTTGATGAAAATGGATCGATCATCC |
| LSAN03845c | Reverse | *TATCCACCTTTACTG* AAGGTTAGCTTTATTCGCATAATAATCGGTCG |
| LSAN06365c | Forward | *TACTTCCAATCCATG* TTGGGCAAAATTTATGGCAAAATTGAAAATGGCAAG |
| LSAN06365c | Reverse | *TATCCACCTTTACTG* AAGGTCAGCTTTATTCGCATAATAATCGGTCGAAG |

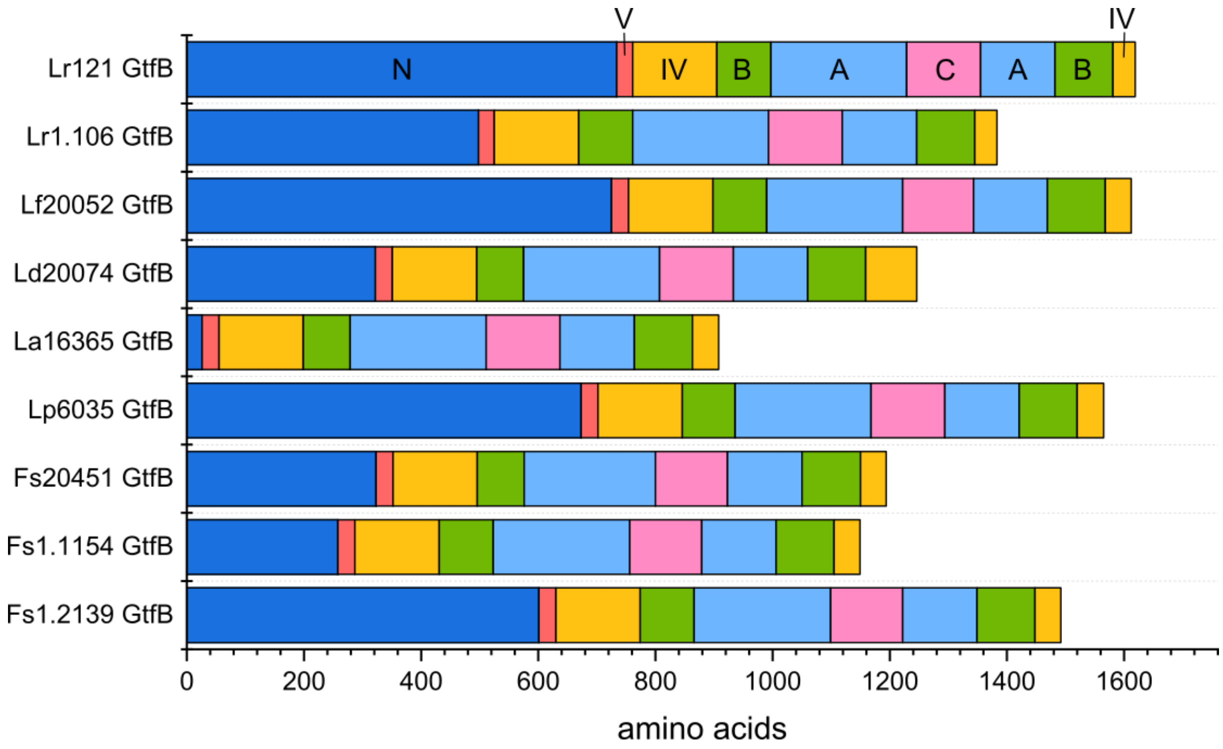


**Fig. S1:** Domain architectures of the GtfB enzymes from different lactic acid bacteria. The strains corresponding to the enzymes are shown in Table 1.

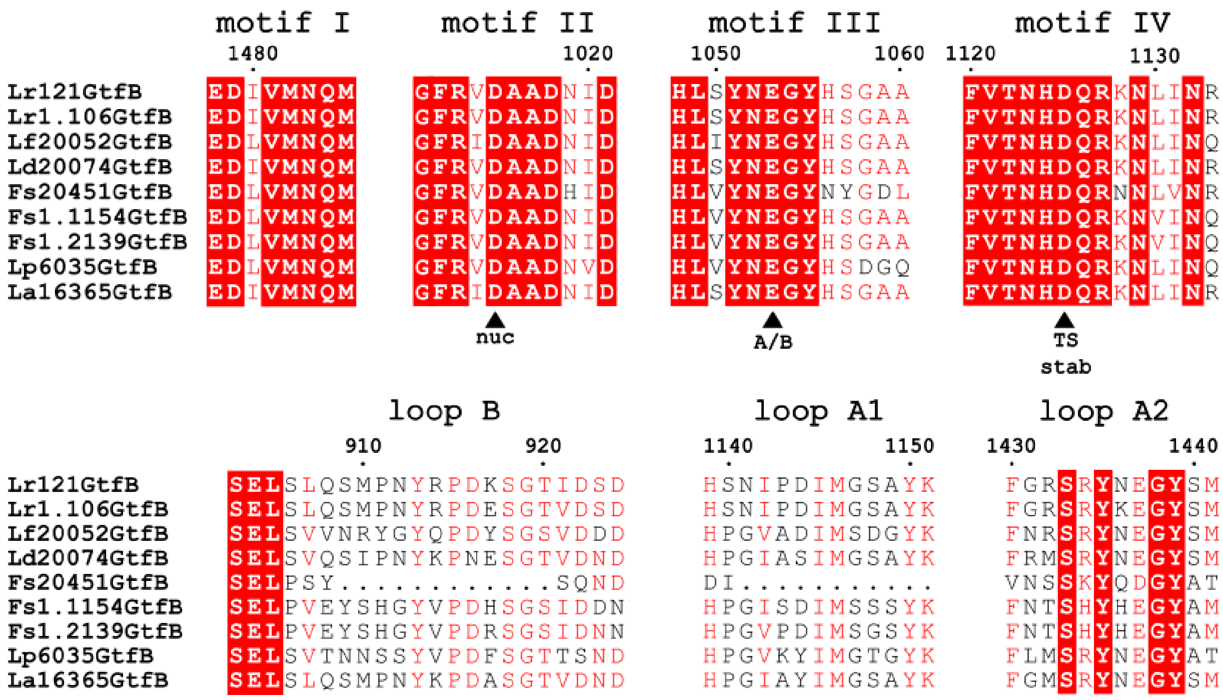


**Fig. S2**: Amino acid sequences of conserved motifs I-IV and loops B, A1 and A2 in the GtfB enzymes used in this study compared to the Lr121 GtfB. The strains corresponding to the enzymes are shown in Table 1.

**Tab. S3**: Sequence identity of the amino acid sequences of GtfBs from the literature and the eight GtfB enzymes investigated in this study in %. The strains corresponding to the enzymes as well as the accession numbers of the sequences used for comparison are shown in Table 1. Sequence identity was determined by using the EMBL-EBI Job Dispatcher sequence analysis tools framework from Clustal Omega.

| | **Lr1.106** | **Lf20052** | **Ld20074** | **La16365** | **Lp6035** | **Fs20451** | **Fs1.1154** | **Fs1.2139** |
|---|---|---|---|---|---|---|---|---|
| **Lr121**[a] | 92.5 | 65.6 | 62.9 | 83.0 | 64.3 | 57.1 | 71.9 | 50.5 |
| **Lr20016**[b] | 56.4 | 70.8 | 59.3 | 72.7 | 54.6 | 81.0 | 84.5 | 81.0 |
| **LrML1**[c] | 96.3 | 65.4 | 62.4 | 82.4 | 65.6 | 57.0 | 71.1 | 50.3 |
| **Lf2970**[d] | 59.1 | 80.6 | 69.1 | 86.4 | 51.3 | 70.6 | 70.4 | 64.9 |
| **Lf3057**[e] | 64.8 | 98.8 | 66.5 | 72.6 | 63.1 | 66.9 | 70.8 | 69.2 |
| **Fs1.1304**[f] | 70.7 | 68.5 | 68.4 | 71.2 | 69.2 | 91.2 | 92.4 | 93.0 |
| **La20655 GtfY**[g] | 51.5 | 56.9 | 50.5 | 59.4 | 47.1 | 51.3 | 58.0 | 47.7 |
| **LrE81**[h] | 48.0 | 36.5 | 32.9 | 39.4 | 46.8 | 32.8 | 37.8 | 30.6 |
| **Lr2613**[i] | 62.9 | 79.0 | 70.1 | 85.7 | 56.3 | 71.7 | 70.3 | 70.2 |
| **St2408**[j] | 79.6 | 63.8 | 66.2 | 84.7 | 62.3 | 57.7 | 69.1 | 49.9 |
| **LrN1**[k] | 80.6 | 73.5 | 83.9 | 84.4 | 68.6 | 68.8 | 68.7 | 68.7 |
| **FsGs2**[l] | 54.8 | 66.9 | 57.4 | 70.2 | 50.6 | 97.6 | 89.5 | 92.7 |
| **La20655 GtfX**[g] | 44.7 | 50.3 | 45.2 | 52.3 | 42.0 | 46.1 | 50.7 | 43.0 |
| **LmL24-B**[m] | 64.1 | 66.4 | 97.6 | 90.0 | 53.9 | 61.7 | 69.6 | 56.8 |
| **WcMBF8-1**[n] | 81.8 | 69.2 | 86.9 | 86.3 | 69.6 | 69.6 | 69.5 | 69.5 |

[a]GtfB from *Limosilactobacillus reuteri* 121[6]
[b]GtfW from *Limosilactobacillus reuteri* DSM 20016[2]
[c]GtfML4 from *Limosilactobacillus reuteri* ML1[7]
[d]GtfB from *Limosilactobacillus fermentum* NCC2790[17]
[e]GtfB from *Limosilactobacillus fermentum* NCC3057[9]
[f]GtfB from *Fructilactobacillus sanfranciscensis* TMW1.1304[10]
[g]GtfX/GtfY from *Ligilactobacillus aviarius* DSM 20655[11]
[h]GtfB from *Limosilactobacillus reuteri* E81[8]
[i]GtfB from *Limosilactobacillus reuteri* NCC2613[12]
[j]GtfB from *Streptococcus thermophilus* NCC2408[14]
[k]GtfB from *Limosilactobacillus reuteri* N1[5]
[l]GtfB from *Fructilactobacillus sanfranciscensis* Gs2[15]
[m]GtfB from *Leuconostoc mesenteroides* L24-B[13]
[n]GtfB from *Weissella confusa* MBF8-1[16]

Numbers correspond to the references in the main text.

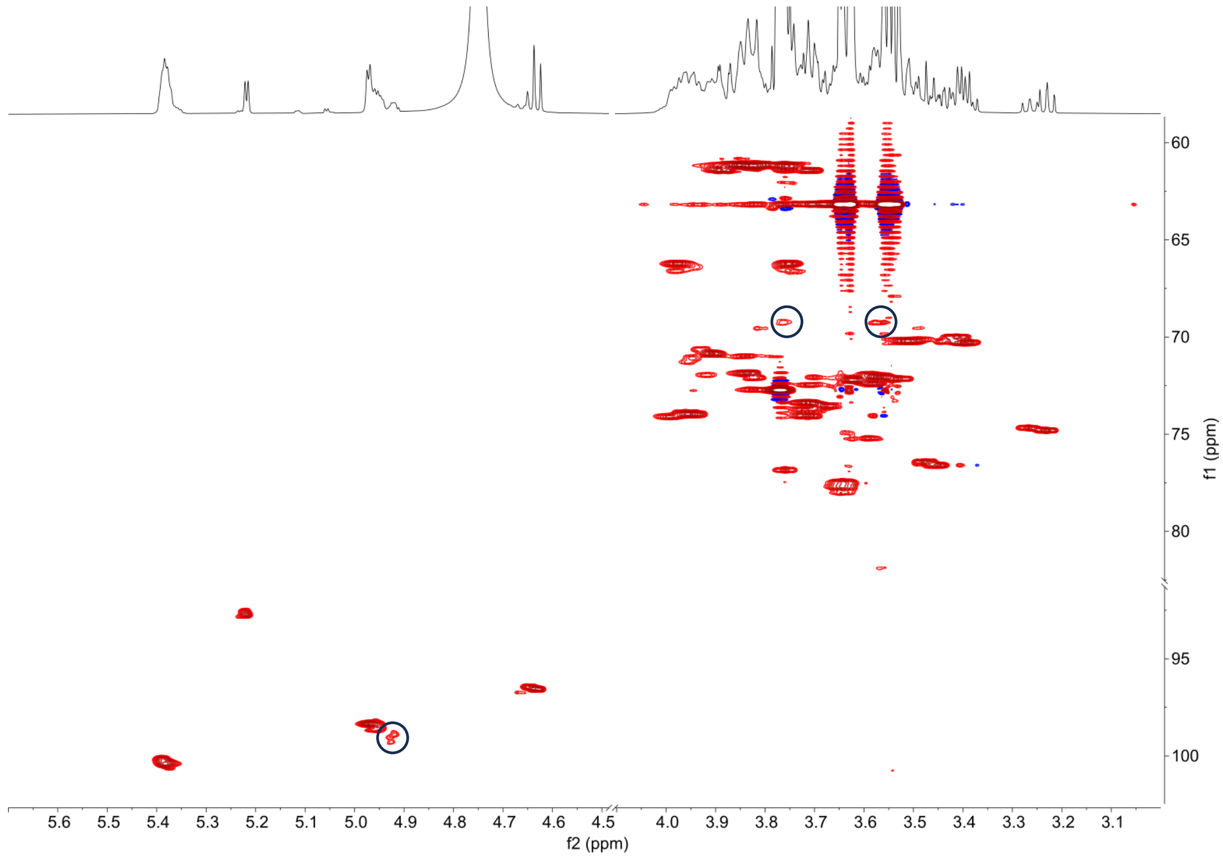


**Fig. S3**: HSQC spectrum of the IMMPs synthesized from maltoheptaose by Lr1.106 GtfB.

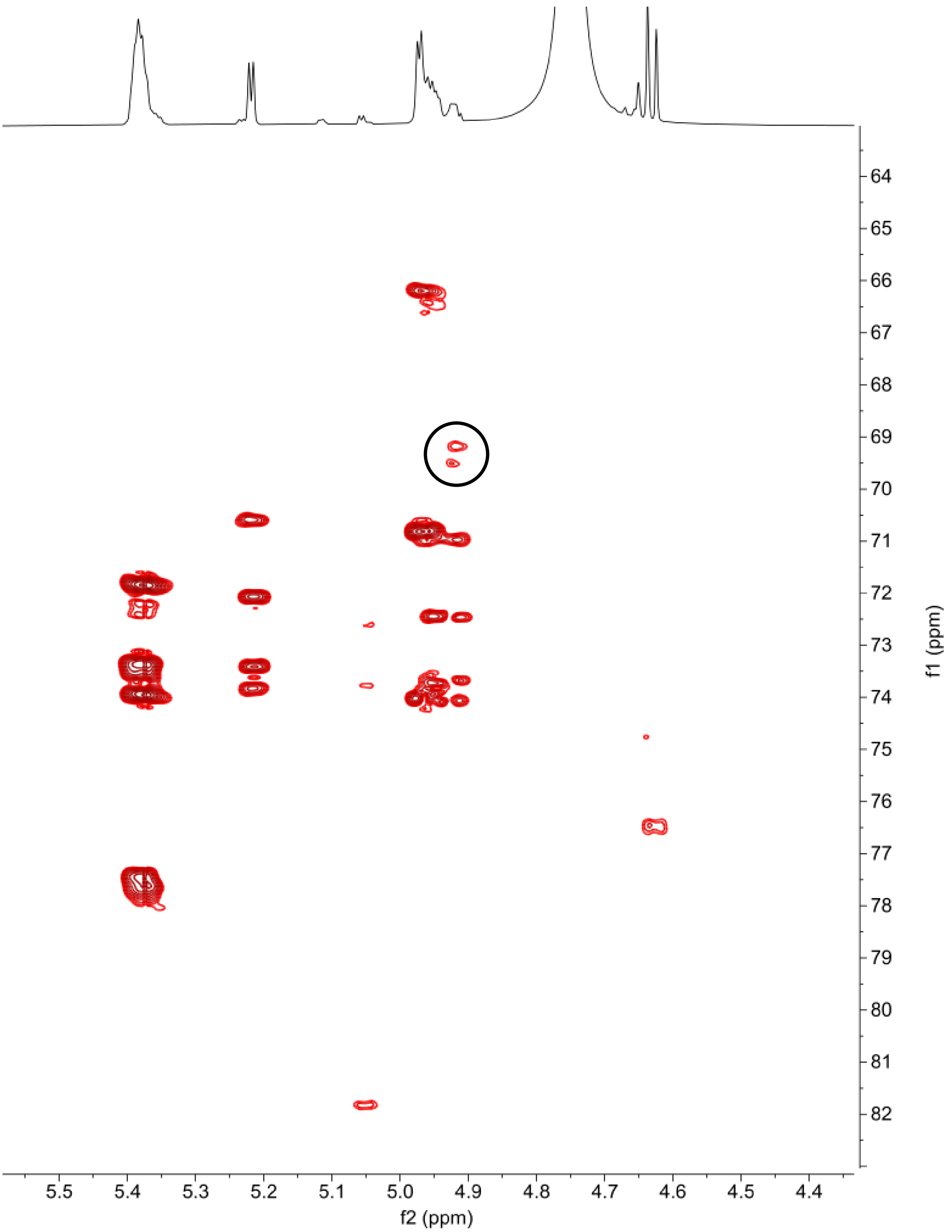


**Fig. S4**: HMBC spectrum of the IMMPs synthesized from maltoheptaose by Lr1.106 GtfB.

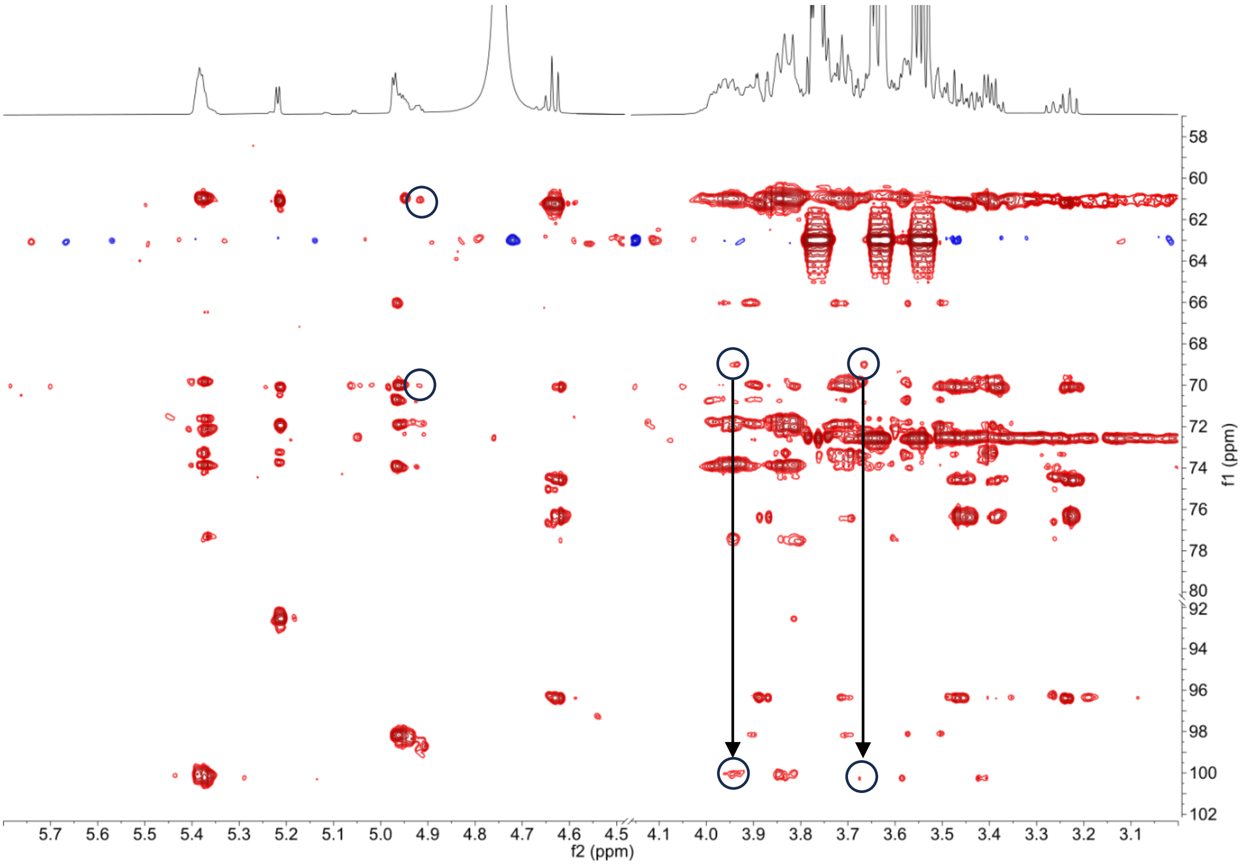


**Fig. S5**: HSQC-TOCSY spectrum of the IMMPs synthesized from maltoheptaose by Lr1.106 GtfB.

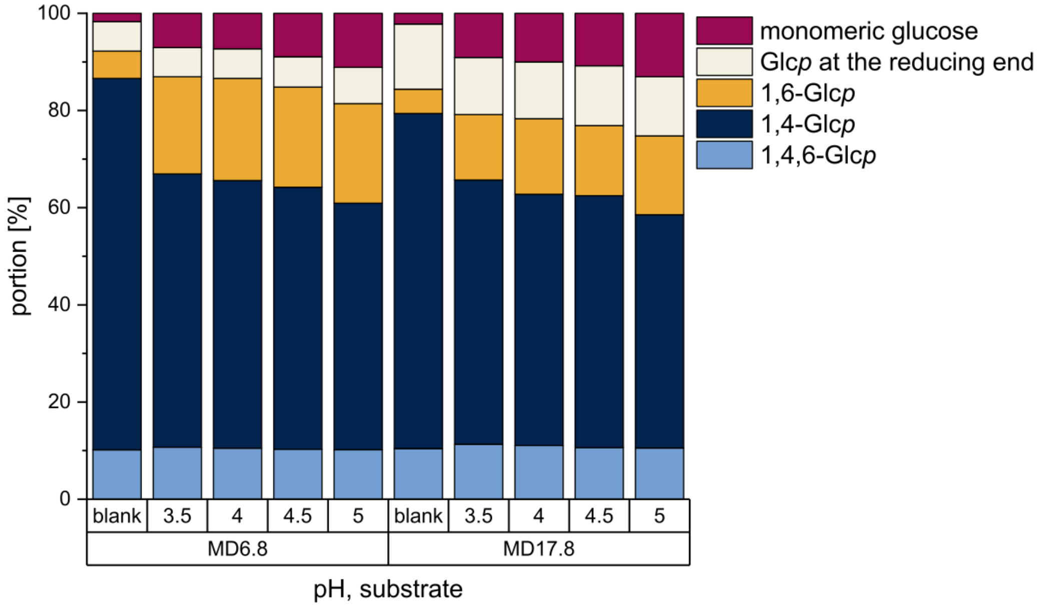


**Fig. S6:** Portions of differently linked glucopyranoses (Glc*p*) and monomeric glucose in the reaction mixtures obtained by the incubation of maltodextrins with a dextrose equivalent of 6.8 (MD6.8) and 17.8 (MD17.8) with the GtfB from *Limosilactobacillus fermentum* DSM 20052 (Lf20052 GtfB). All samples were incubated at 37 °C and different pH values for 24 h. The structural composition of the mixtures was analyzed by $^{1}$H NMR spectroscopy.

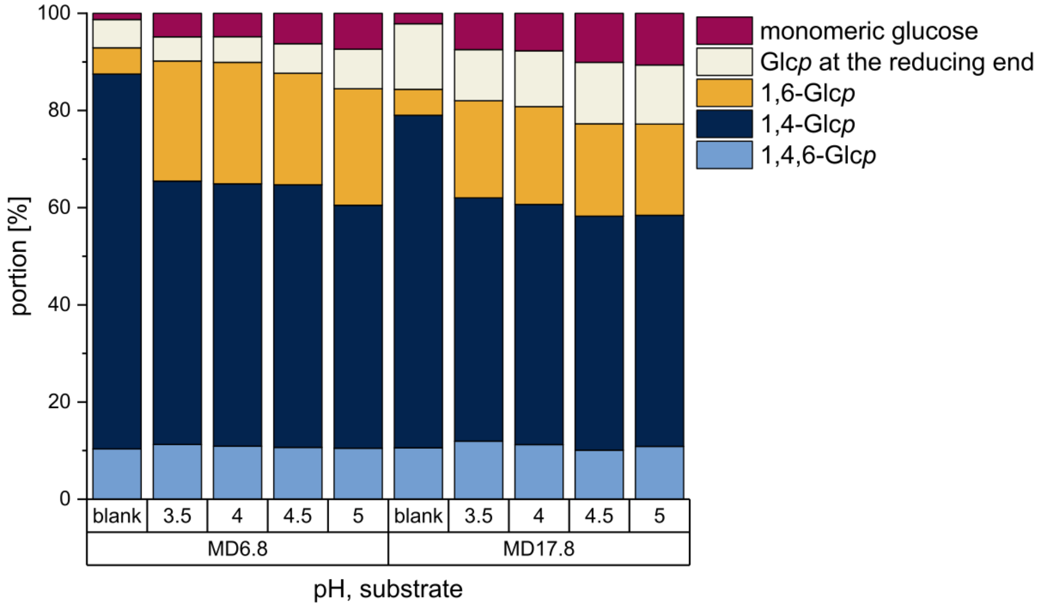


**Fig. S7**: Portions of differently linked glucopyranoses (Glc*p*) and monomeric glucose in the reaction mixtures obtained by the incubation of maltodextrins with a dextrose equivalent of 6.8 (MD6.8) and 17.8 (MD17.8) with the GtfB from *Limosilactobacillus delbrueckii* subsp. *delbrueckii* DSM 20074 (Ld20074 GtfB). All samples were incubated at 37 °C and different pH values for 24 h. The structural composition of the mixtures was analyzed by $^{1}$H NMR spectroscopy.

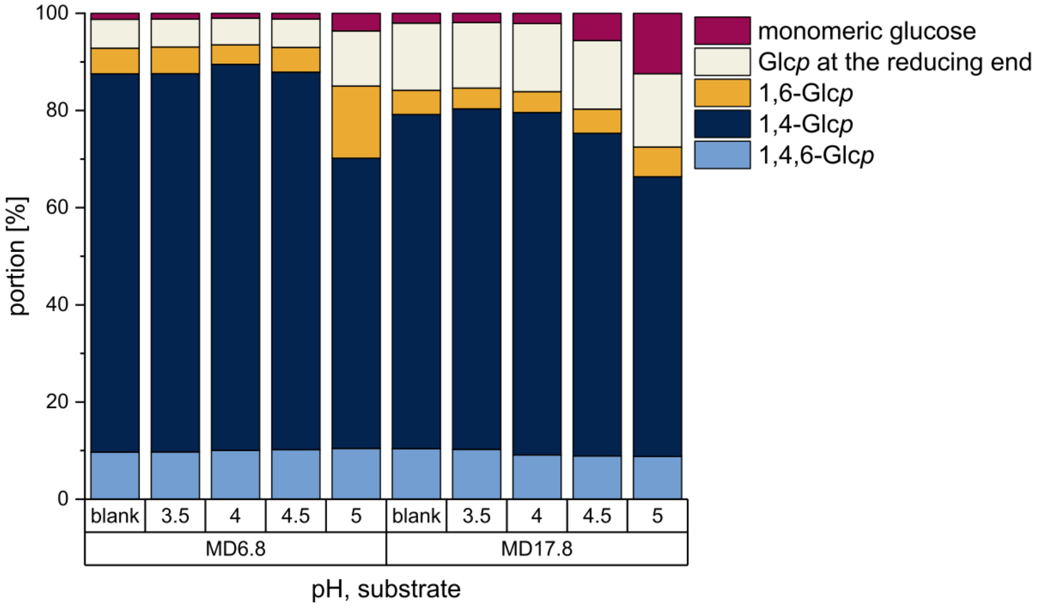


**Fig. S8**: Portions of differently linked glucopyranoses (Glc*p*) and monomeric glucose in the reaction mixtures obtained by the incubation of maltodextrins with a dextrose equivalent of 6.8 (MD6.8) and 17.8 (MD17.8) with the GtfB from *Lactiplantibacillus argentoratensis* DSM 16365 (La16365 GtfB). All samples were incubated at 37 °C and different pH values for 24 h. The structural composition of the mixtures was analyzed by $^{1}$H NMR spectroscopy.

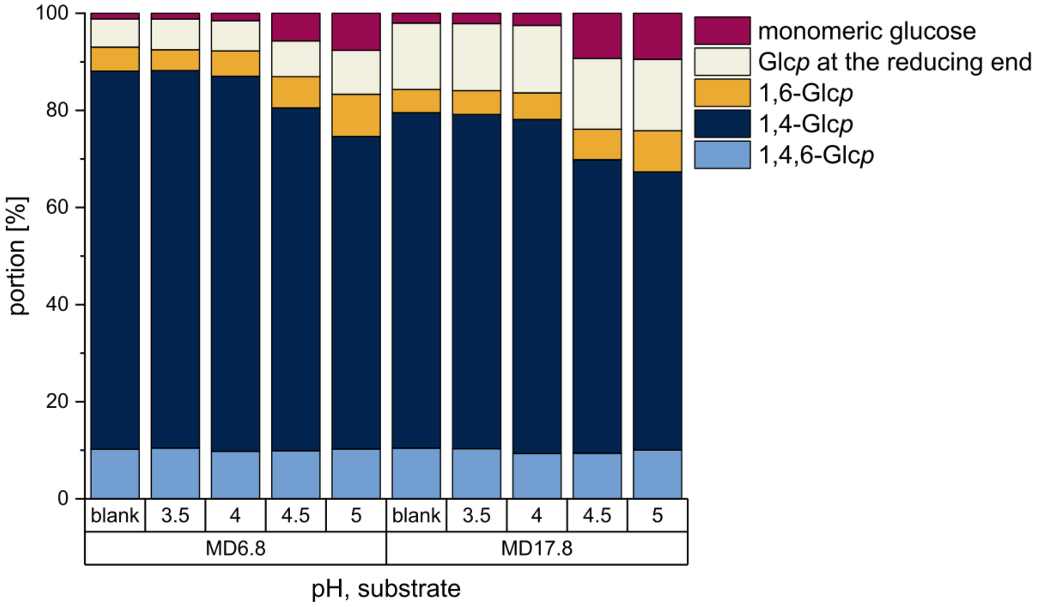


**Fig. S9:** Portions of differently linked glucopyranoses (Glc*p*) and monomeric glucose in the reaction mixtures obtained by the incubation of maltodextrins with a dextrose equivalent of 6.8 (MD6.8) and 17.8 (MD17.8) with the GtfB from *Limosilactobacillus panis* DSM 6035 (Lp6035 GtfB). All samples were incubated at 37 °C and different pH values for 24 h. The structural composition of the mixtures was analyzed by $^{1}$H NMR spectroscopy.

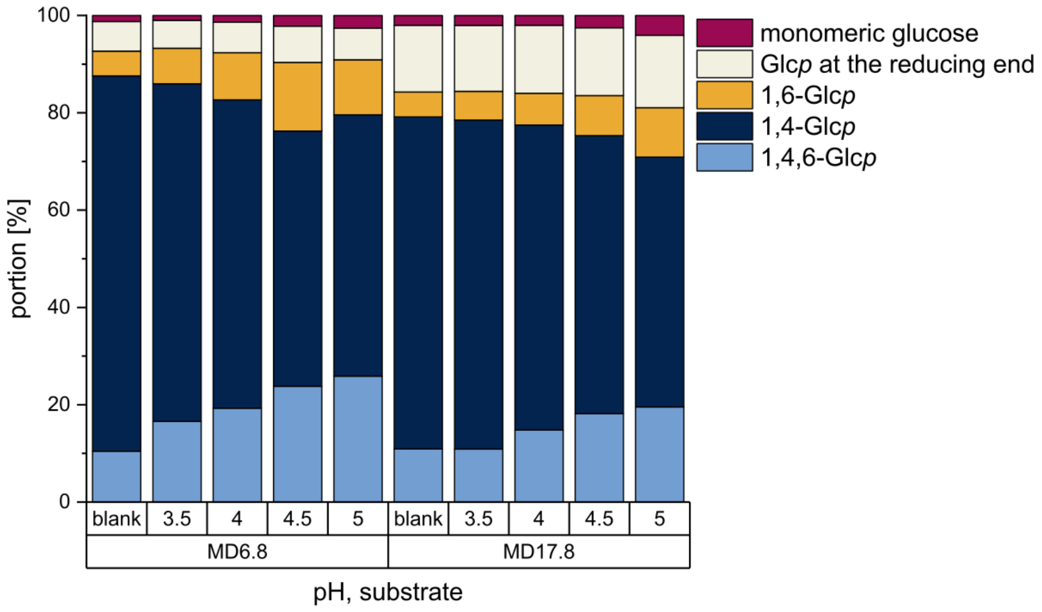


**Fig. S10**: Portions of differently linked glucopyranoses (Glc*p*) and monomeric glucose in the reaction mixtures obtained by the incubation of maltodextrins with a dextrose equivalent of 6.8 (MD6.8) and 17.8 (MD17.8) with the GtfB from *Fructilactobacillus sanfranciscensis* DSM 20451 (Fs20451 GtfB). All samples were incubated at 37 °C and different pH values for 24 h. The structural composition of the mixtures was analyzed by $^{1}$H NMR spectroscopy.

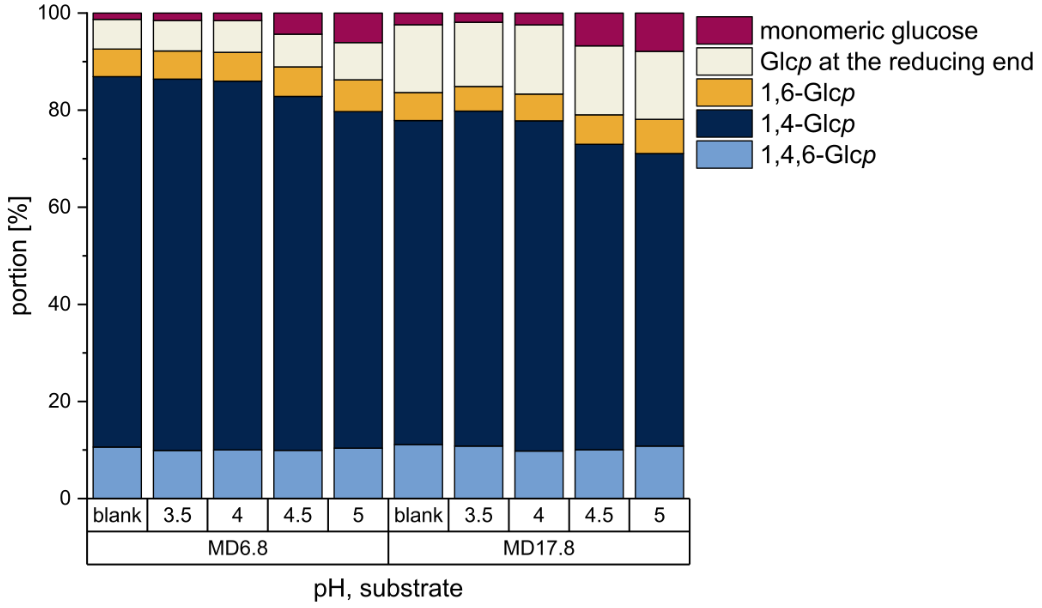


**Fig. S11**: Portions of differently linked glucopyranoses (Glc*p*) and monomeric glucose in the reaction mixtures obtained by the incubation of maltodextrins with a dextrose equivalent of 6.8 (MD6.8) and 17.8 (MD17.8) with the GtfB from *Fructilactobacillus sanfranciscensis* TMW 1.1154 (Fs1.1154 GtfB). All samples were incubated at 37 °C and different pH values for 24 h. The structural composition of the mixtures was analyzed by $^{1}$H NMR spectroscopy.

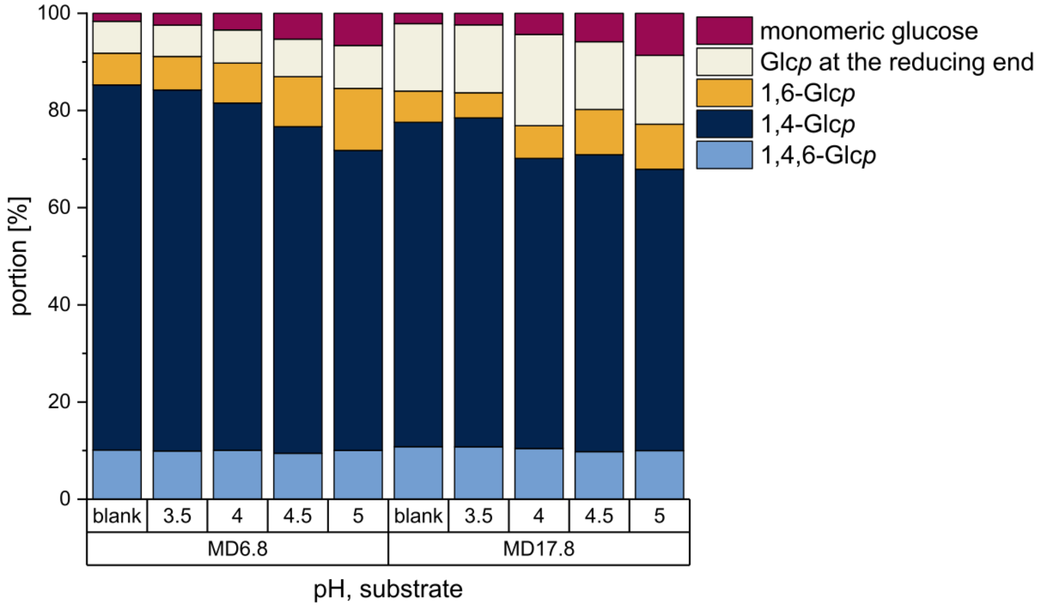


**Fig. S12**: Portions of differently linked glucopyranoses (Glc*p*) and monomeric glucose in the reaction mixtures obtained by the incubation of maltodextrins with a dextrose equivalent of 6.8 (MD6.8) and 17.8 (MD17.8) with the GtfB from *Fructilactobacillus sanfranciscensis* TMW 1.2139 (Fs1.2139 GtfB). All samples were incubated at 37 °C and different pH values for 24 h. The structural composition of the mixtures was analyzed by $^{1}$H NMR spectroscopy.

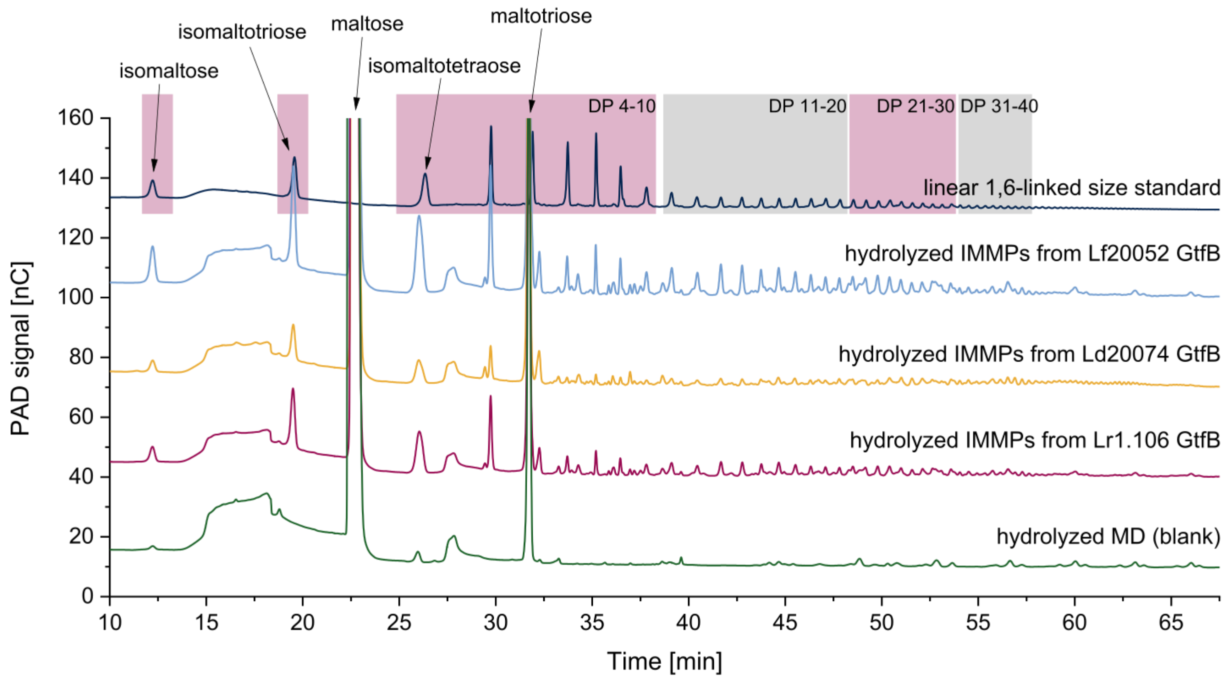


**Fig. S13**: HPAEC-PAD chromatograms of the size standard (partially hydrolyzed linear dextran, dark blue), the hydrolyzed isomalto/malto-polysaccharides (IMMPs) synthesized by the GtfBs from *Limosilactobacillus* (*Llb.*) *fermentum* DSM 20052 (Lf20052 GtfB, light blue), *Lactobacillus delbrueckii* subsp. *delbrueckii* DSM 20074 (LD20074 GtfB, yellow), and *Llb. reuteri* TMW 1.106 (Lr1.106 GtfB, red) from maltodextrin with a dextrose equivalent of 6.8, and the hydrolyzed maltodextrin with a dextrose equivalent of 6.8 (MD, green). The degrees of polymerization (DP) of the size standard peaks are marked with red and gray boxes.

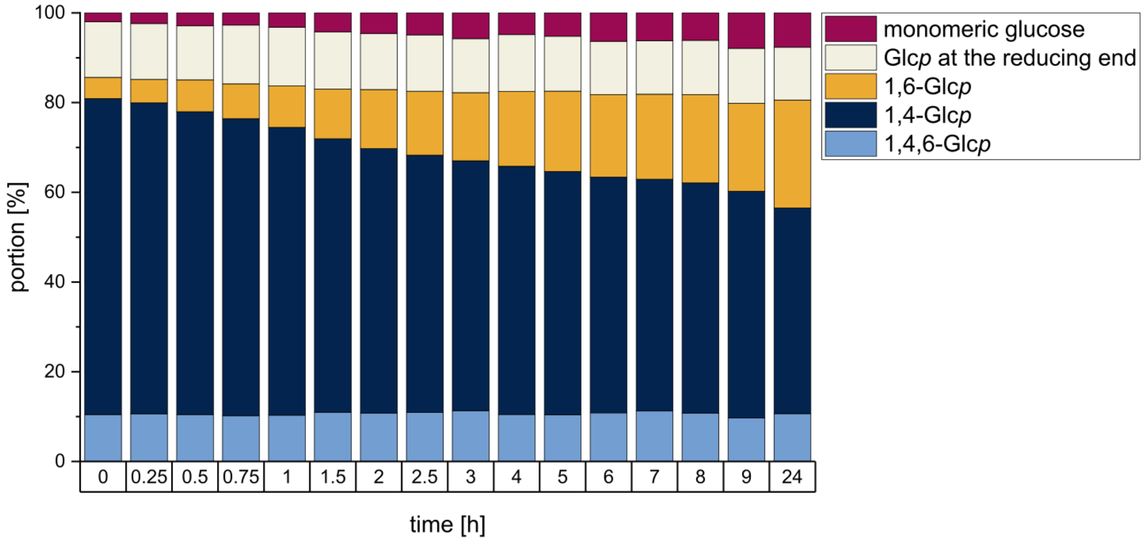


**Fig. S14**: Portions of differently linked glucopyranoses (Glc*p*) and monomeric glucose at different times during the conversion of maltodextrin with a dextrose equivalent of 17.8 (MD17.8) by the GtfB from *Lactobacillus delbrueckii* subsp. *delbrueckii* DSM 20074. The reaction was carried out at pH 4.5 and 37 °C. The structural composition of the mixtures was analyzed by $^{1}$H NMR spectroscopy.

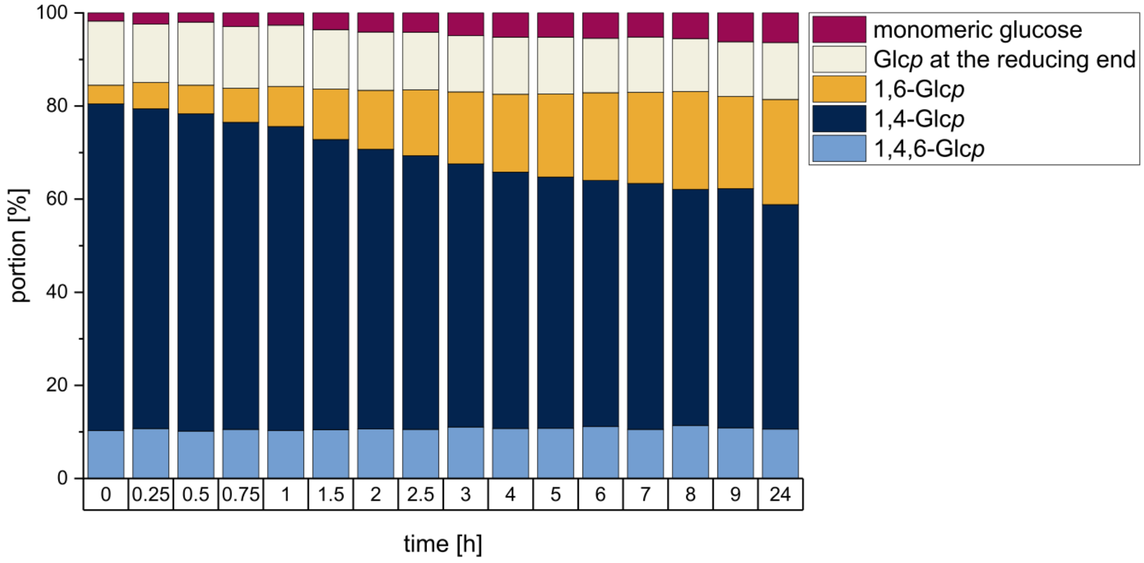


**Fig. S15**: Portions of differently linked glucopyranoses (Glc*p*) and monomeric glucose at different times during the conversion of maltodextrin with a dextrose equivalent of 17.8 (MD17.8) by the GtfB from *Limosilactobacillus fermentum* DSM 20052. The reaction was carried out at pH 4.5 and 37 °C. The structural composition of the mixtures was analyzed by $^{1}$H NMR spectroscopy.

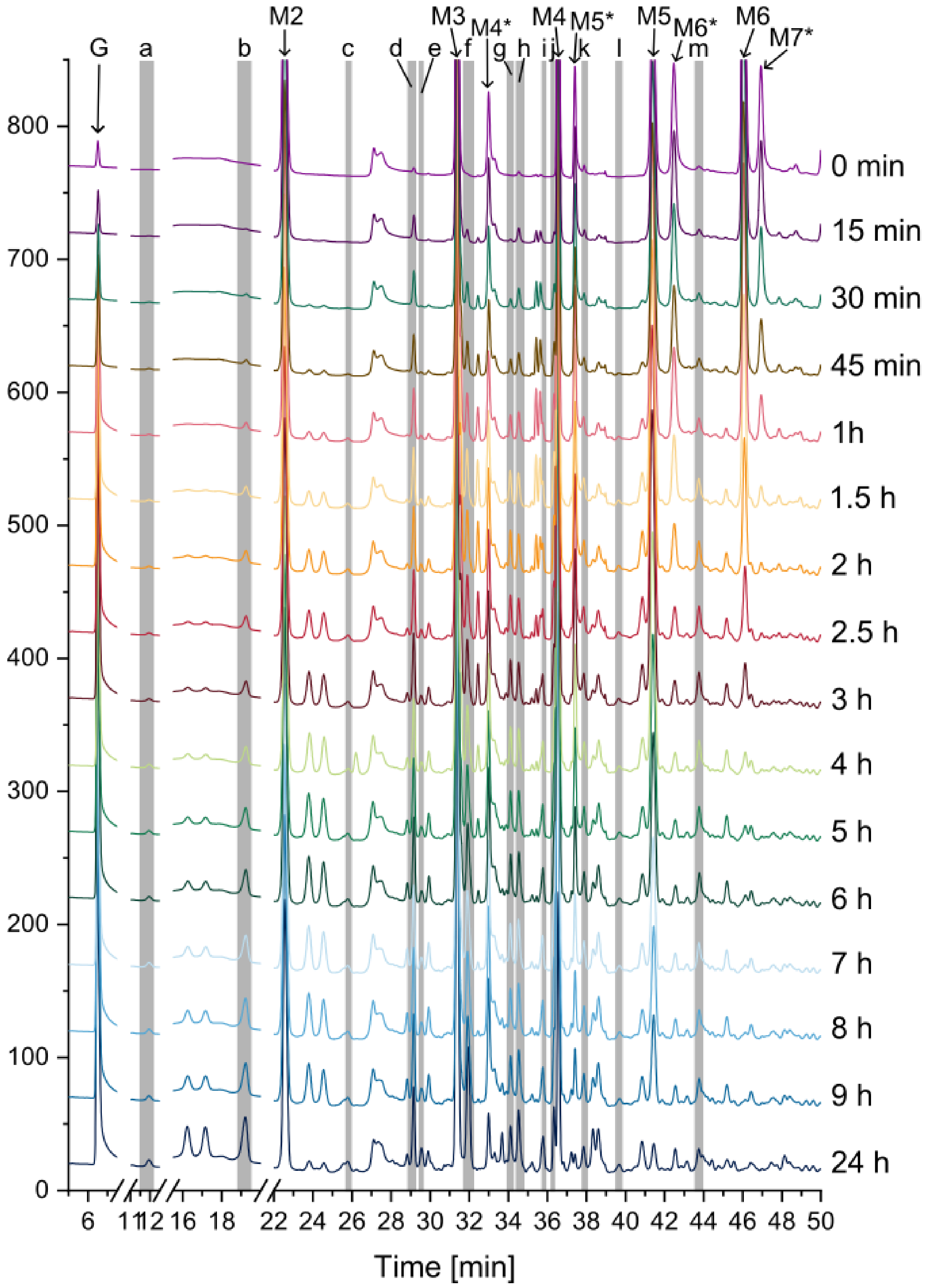


**Fig. S16**: HPAEC chromatograms of samples taken at different times during IMMP synthesis by Lr1.106 GtfB from maltodextrin with a dextrose equivalent of 17.8. G = glucose, M2 = maltose, M3 = maltotriose, M4 = maltotetraose, M5 = maltopentaose, M6 = maltohexaose, M4*-M7* = limit dextrins with the respective degree of polymerization. The molecular structures of a – m are shown in Fig. S19.

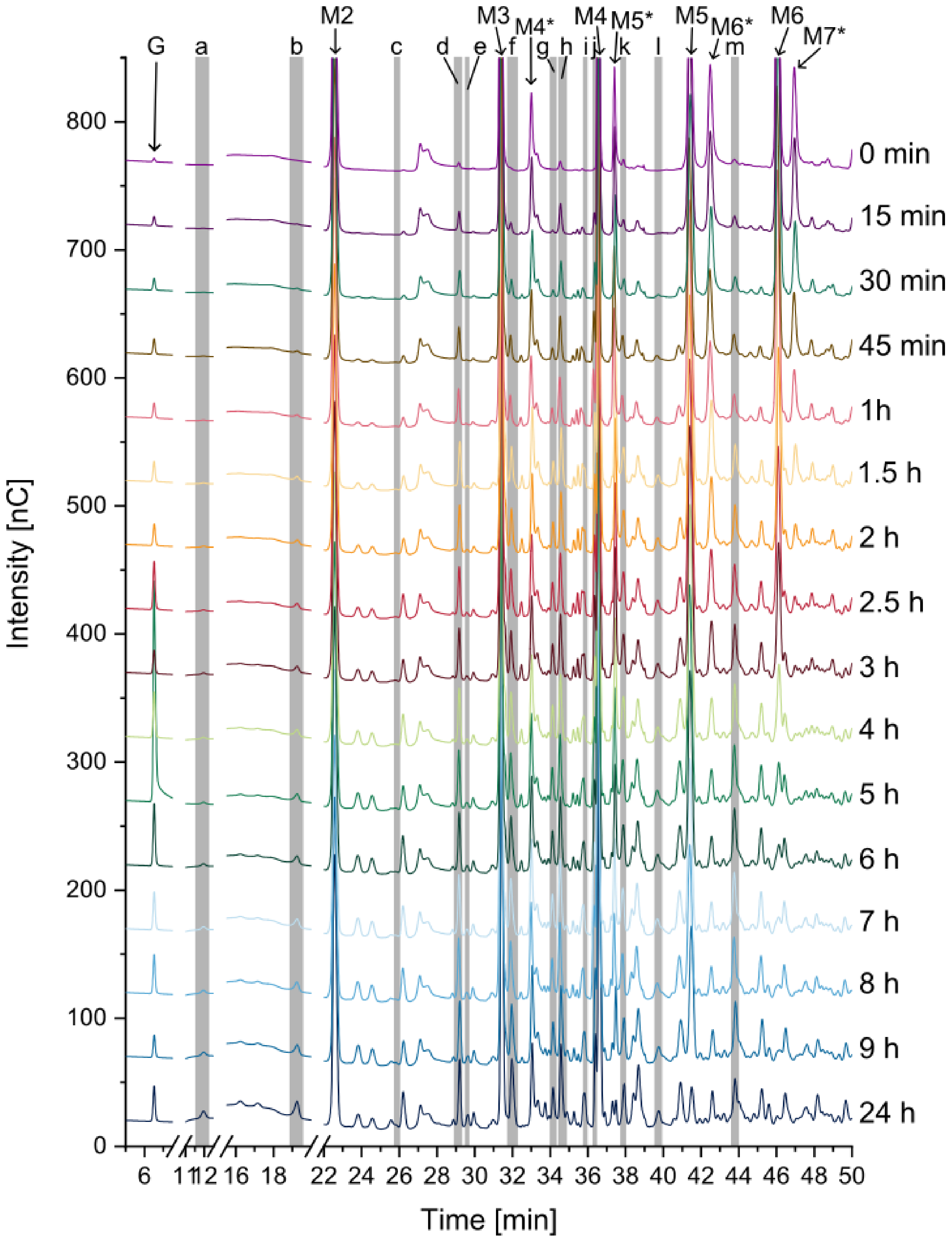


**Fig. S17**: HPAEC chromatograms of samples taken at different times during IMMP synthesis by Lf20052 GtfB from maltodextrin with a dextrose equivalent of 17.8. G = glucose, M2 = maltose, M3 = maltotriose, M4 = maltotetraose, M5 = maltopentaose, M6 = maltohexaose, M4*-M7* = limit dextrins with the respective degree of polymerization. The molecular structures of a – m are shown in Fig. S19.

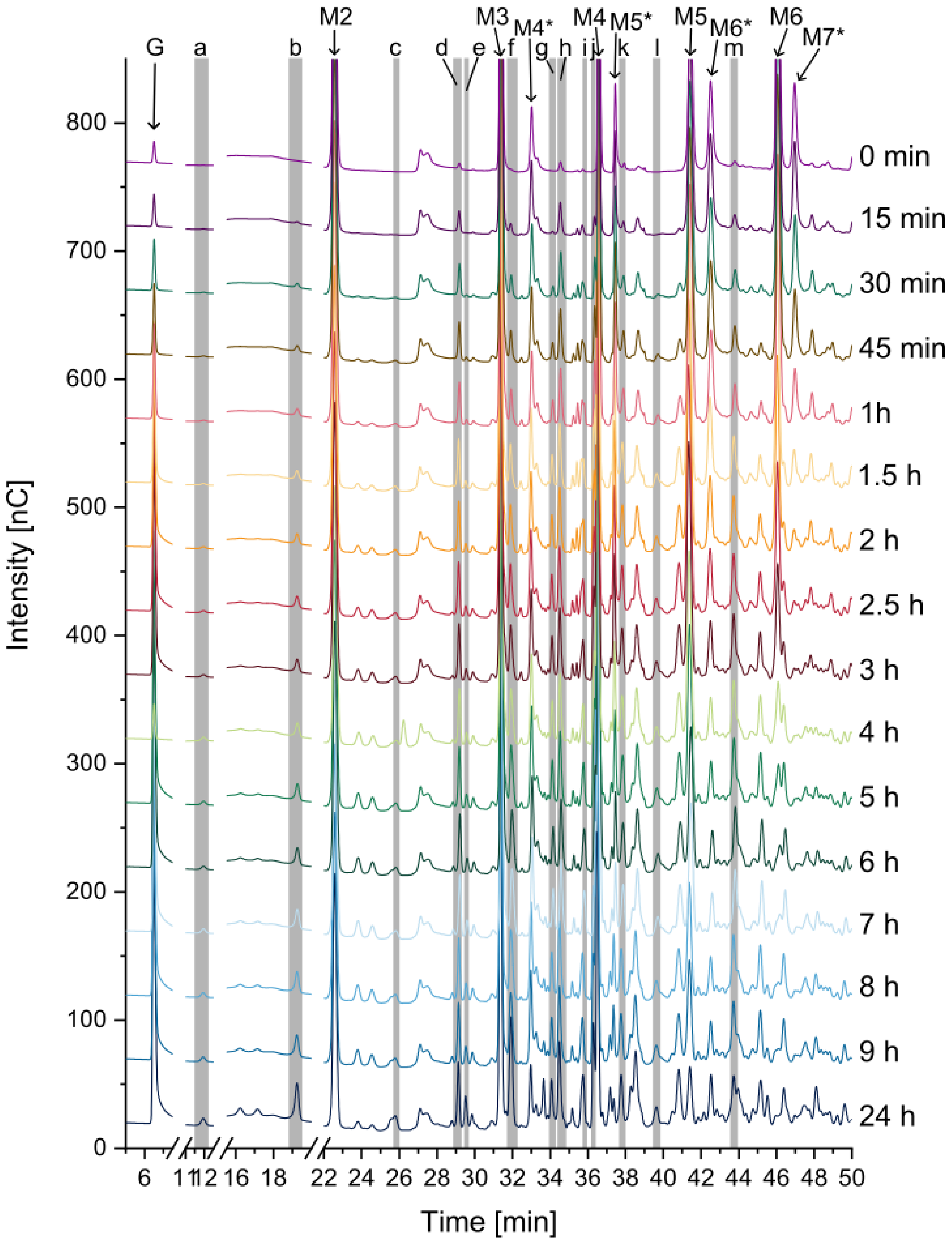


**Fig. S18**: HPAEC chromatograms of samples taken at different times during IMMP synthesis by Ld20074 GtfB from maltodextrin with a dextrose equivalent of 17.8. G = glucose, M2 = maltose, M3 = maltotriose, M4 = maltotetraose, M5 = maltopentaose, M6 = maltohexaose, M4*-M7* = limit dextrins with the respective degree of polymerization. The molecular structures of a – m are shown in Fig. S19.

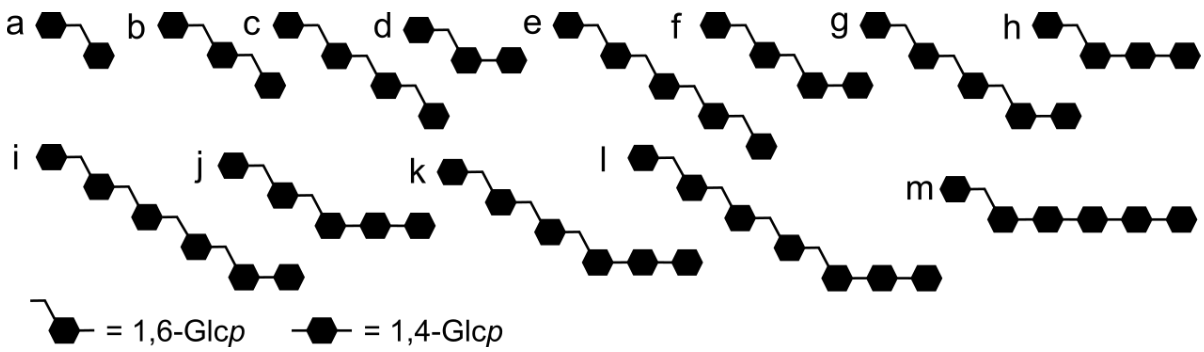


**Fig. S19**: Molecular structures of different IMMP standards that were synthesized by the dextransucrase of *Ligilactobacillus animalis* TMW 1.971 from different malto-oligosaccharides.
